# Metabolomic profiling reveals altered phenylalanine metabolism in Parkinson’s disease in an Egyptian cohort

**DOI:** 10.3389/fmolb.2024.1341950

**Published:** 2024-03-07

**Authors:** Nourhan Shebl, Shaimaa El-Jaafary, Ayman A. Saeed, Passent Elkafrawy, Amr El-Sayed, Samir Shamma, Rasha Elnemr, Jaidaa Mekky, Lobna A. Mohamed, Omar Kittaneh, Hassan El-Fawal, Mie Rizig, Mohamed Salama

**Affiliations:** ^1^ Institute of Global Health and Human Ecology (I-GHHE), The American University in Cairo, Cairo, Egypt; ^2^ Neurology Department, Faculty of Medicine, Cairo University, Giza, Egypt; ^3^ Global Brain Health Institute (GBHI), Trinity College Dublin, Dublin, Ireland; ^4^ Applied Organic Chemistry Department, Chemical Industries Research Institute, National Research Centre (NRC), Giza, Egypt; ^5^ Technology and Energy Research Center, Effat University-College of Engineering-NSMTU, Jeddah, Saudi Arabia; ^6^ Social Research Center, The American University in Cairo, Cairo, Egypt; ^7^ Climate Change Information Center & Expert Systems (CCICES), Agriculture Research Center, Giza, Egypt; ^8^ Neurology Department, Faculty of Medicine, Alexandria University, Alexandria, Egypt; ^9^ Queen Square, Institute of Neurology, University College London, London, United Kingdom; ^10^ Faculty of Medicine, Mansoura University, Mansoura, Egypt

**Keywords:** trans-cinnamate, phenylalanine, dopamine, tyrosine, metabolomics, PD, PAL, PAH

## Abstract

**Introduction:** Parkinson’s disease (PD) is the most common motor neurodegenerative disease worldwide. Given the complexity of PD etiology and the different metabolic derangements correlated to the disease, metabolomics profiling of patients is a helpful tool to identify patho-mechanistic pathways for the disease development. Dopamine metabolism has been the target of several previous studies, of which some have reported lower phenylalanine and tyrosine levels in PD patients compared to controls.

**Methods:** In this study, we have collected plasma from 27 PD patients, 18 reference controls, and 8 high-risk controls to perform a metabolomic study using liquid chromatography-electrospray ionization–tandem mass spectrometry (LC-ESI-MS/MS).

**Results:** Our findings revealed higher intensities of trans-cinnamate, a phenylalanine metabolite, in patients compared to reference controls. Thus, we hypothesize that phenylalanine metabolism has been shifted to produce trans-cinnamate via L-phenylalanine ammonia lyase (PAL), instead of producing tyrosine, a dopamine precursor, via phenylalanine hydroxylase (PAH).

**Discussion:** Given that these metabolites are precursors to several other metabolic pathways, the intensities of many metabolites such as dopamine, norepinephrine, and 3-hydroxyanthranilic acid, which connects phenylalanine metabolism to that of tryptophan, have been altered. Consequently, and in respect to *Metabolic Control Analysis* (MCA) theory, the levels of tryptophan metabolites have also been altered. Some of these metabolites are tryptamine, melatonin, and nicotinamide. Thus, we assume that these alterations could contribute to the dopaminergic, adrenergic, and serotonergic neurodegeneration that happen in the disease.

## Introduction

PD is a complex progressive neurodegenerative disorder and the most common movement disorder globally. The cardinal signs of PD involve motor symptoms such as tremors, bradykinesia/akinesia, postural instability, and rigidity. Additionally, PD is usually accompanied by non-motor symptoms such as autonomic nervous system dysfunction (orthostatic hypotension and obstipation), cognition impairment, mood disorders, and/or sleep problems (rapid-eye- movement- REM, sleep behavior disorder, insomnia, or daytime sleepiness) (Peball et al., 2020). Although, pathologically characterized by the loss of the dopaminergic neurons in the midbrain, PD pathology affects other sites that includes non-dopaminergic neurons (Simon et al., 2020). For instance, Braak’s hypothesis states that PD starts with a pathogenic entrance in the olfactory bulb (OB), which stimulates the pathology of alpha-synuclein (α-syn) in OB and dorsal motor nucleus of the vagus (DMV), after which it invades the brain and cause the neurodegeneration in the dopaminergic neurons in the SN (Borghammer et al., 2022).

What triggers the death of dopaminergic neurons, has been the focus of several research activities. That is why the synthesis of dopamine from its precursor, phenylalanine, have been widely studied (Marchiosi et al., 2020). Typically, the first 2 steps in the dopamine synthesis take place in the cytosol of catecholaminergic neurons. After the conversion of phenylalanine to L-tyrosine via PAH, tyrosine is, then, hydroxylated by tyrosine hydroxylase (TH) to produce L-DOPA. BH4 (tetrahydrobiopterin) strongly regulates this oxidation step as a cofactor, which is produced by guanosine triphosphate (GTP) by GTP cyclohydrolase (GPTCH). Then, aromatic amino acid decarboxylase (AAAD) or as commonly known DOPA decarboxylase, decarboxylases DOPA to yield dopamine (Meiser et al., 2013).

Phenylalanine is an essential amino acid that cannot be produced in the body and must be supplemented in diet. It is integrated into synthesizing many proteins, catecholamines, and melanin. One of its leading roles is being the precursor of the amino acid Tyrosine and, subsequently, L-dopa and the neurotransmitters, dopamine and norepinephrine (Kohlmeier, 2003). Two main pathways have been identified for phenylalanine’s kinetics in humans. The first one is for the irreversible degradation of phenylalanine through its hydroxylation by PAH, which is considered the rate-limiting step in dopamine synthesis, to yield L-tyrosine. The second pathway is through the transamination of phenylalanine to produce phenylpyruvate that is followed by several metabolic phases which produce many metabolites such as phenylacetate, phenyl lactate, and o-hydroxyphenylacetate (Kaufman, 1999). However, several studies found that phenylalanine can be metabolized via 6 degradation pathways, including degradation by L-phenylalanine ammonia lyase (PAL) through the phenylpropanoids pathway (Oliphant and Allen-Vercoe, 2019; Genome, 2023a). PAL metabolizes phenylalanine into 2 products: trans-cinnamate and ammonia (Sarkissian et al., 1999). Trans-cinnamate is a metabolite that exists in all living organisms, ranging from bacteria to humans (Genome, 2023a; HMDB, 2023).

In phenylketonuria (PKU), a condition characterized by the significant reduction in the activity of PAH and the accumulation of phenylalanine, synthetic PAL pills were used to enhance the metabolism of phenylalanine into trans-cinnamate (Sarkissian et al., 1999). A recent animal study on rats suffering from PKU showed that genetic transfer of PAH and PAL, both phenylalanine and neurotransmitters were positively altered with significant improvement in the animal behavior (Manek et al., 2021).

Ammonia, the by-product of phenylalanine metabolism into trans-cinnamate via PAL, is considered a neurotoxic agent (Bobermin et al., 2015). Ammonia has been shown to increase the oxidative stress in neurons, which has been proven to increase neurodegeneration in PD (Chang and Chen, 2020). Moreover, ammonia showed the ability to inhibit the consumption of mitochondrial oxygen, which is considered to be a risk factor for developing many neurodegenerative disorders (Oliphant and Allen-Vercoe, 2019).

Identifying alterations at the level of metabolomes offers a better understanding of disease process (Kapoore and Vaidyanathan, 2016). Metabolomics has been studied several times in PD; for instance, one study using 49 non-treated PD patients’ plasma yielded a strong prediction of PD progression (LeWitt et al., 2017). Another non-targeted metabolomics study on 39 preclinical PD patients study showed alterations in 33 metabolites’ levels, including significant reductions in the levels of the free fatty acids (FFAs), suggesting alterations in FFAs metabolism, oxidative stress, and mitochondrial dysfunction; these results were further validated by a targeted HPLC-QqQ-Ms approach (Gonzalez-Riano et al., 2021). A recent study using non-targeted metabolomics analysis on 30 PD patients revealed alterations in the levels of 30 metabolites, including the metabolism of lipids and lipid-like molecules (Lan et al., 2023).

**FIGURE 1:**
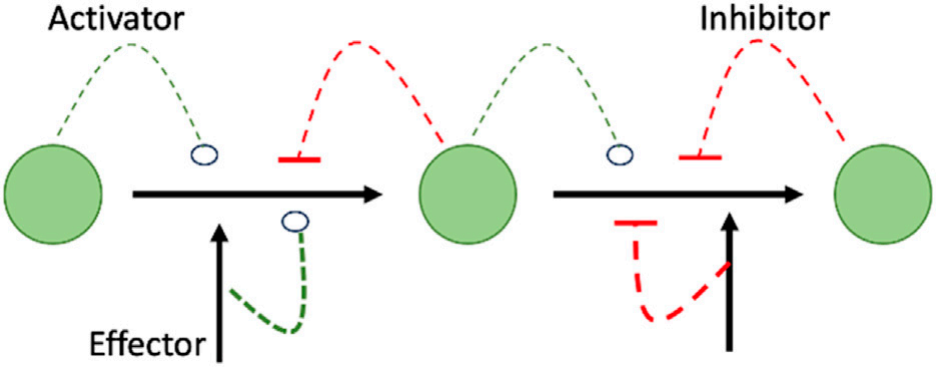
Shows that summary of MCA theory highlighting the interrelationship between effector and downstream events of increase or decrease of metabolites as it shows how the concentration of an activator, inhibitor, or effector can affect the expression/activity of the following enzyme in the metabolic pathway, resulting in decrease/increase of the concentration of the following metabolite in a cascade manner.

The value of studying metabolomics is not only to identify changes in levels of metabolites, but also, in studying pathways that can be affected by the metabolic changes as per the Metabolic Control Theory (MCA). MCA explains the meaning of elasticity coefficients by elaborating how the concentration of one substrate, product, or effector can affect the expression and the flow of the entire pathway (Figure 1) (Moreno-Sánchez et al., 2008).

Adopting this pathways approach, the present metabolomic study focused on the metabolism of phenylalanine through L-phenylalanine ammonia lyase (PAL) and phenylalanine hydroxylase (PAH), and their possible relationship with L-tryptophan and its metabolites in PD patients, compared to reference, non-PD, controls.

## Materials and methods

### Ethical aspects

Ethical approval was obtained from the responsible Institutional Review Board/AUC-IRB (Ethics Approval # 2021-2022-058 and 2021-2022-203). Only subjects who provided written informed consent were included in the study.

### Patients recruitment

Twenty seven patients (55.8 ± 10.7 years old (s.d.); 19 males and 8 females) were recruited from Kasr El-Ainy Hospital and Alexandria University Hospital, Egypt. Neurologists diagnosed the patients according to the United Kingdom PD Society Brain Bank criteria (Clarke et al., 2016). Patients with psychiatric illness, usage of antipsychotics, endocrinal or metabolic disturbances such as thyroid disturbances or diabetes, and/or other forms of parkinsonism like multiple system atrophy (MSA) were excluded from the study, noting that all the recruited patients were on L-DOPA therapy.

### Reference controls recruitment

Eighteen reference controls (52.3 ± 11.9 years old (s.d.); 12 males and 6 females) were recruited by a neurologist. Reference controls were recruited after signing a consent of approval, noting that any volunteered individual with psychiatric illness, usage of antipsychotics, endocrinal or metabolic disturbances such as thyroid disturbances or diabetes, and/or other forms of parkinsonism like MSA were excluded from the study.

### High risk controls

Additional, 8 healthy controls who were assessed, initially, as clinically non-PD-patients, started to show prodromal manifestations during follow up visits. For that, we highlighted this group as a high-risk control group.

### Plasma collection and processing

1 mL of blood was collected by phlebotomy in a heparin blood-collecting tube. The collection was done between 10 and 12 a.m. The heparin tubes were centrifuged at 2000 *g* for 10 min; then, the plasma was aspirated and transferred into 1.5 mL Eppendorf tubes, and DMSO was added by volume ratio of 50 ul for each 1 mL plasma. Finally, the plasma was stored in a −80 freezer until the metabolomics analysis was performed.

### Equipment

The analysis of the sample was performed using LC-ESI-MS/MS with an ExionLC AC system for separation and SCIEX Triple Quad 5,500+ MS/MS system equipped with an electrospray ionization (ESI) for detection.

### Positive mode

Frozen plasma samples were thawed at room temperature, vortexed and incubated for 10 min on ice. The samples were then centrifuged at 10,000 × g for 10 min at 4°C. The supernatant was transferred and filtered through 0.2 μm filter syringe before injection (Xiao et al., 2012). The separation was performed with a Ascentis^®^ C18 Column (4.6 × 150 mm, 3 µm). The mobile phases consisted of two eluents A: 0.1% formic acid; B: acetonitrile (LC grade). The mobile phase gradient was programmed as follows: 10% B at 0–1 min, 10%–90% B from 1–21 min, 90% B from 21–25 min, 10% at 25.1, 10% from 25.1–28 min. The flow rate was 0.7 mL/min, and the injection volume was 10 µL. For MS/MS analysis, positive ionization mode was applied with a scan (EMS-IDA-EPI) from 100 to 1000 DA for MS1 with the following parameters: curtain gas: 25 psi; Ion-Spray voltage: 4,500; source temperature: 500°C; ion source gas 1 & 2 were 45 psi and from 50 to 800 DA for MS2 with a de-clustering potential: 80; collision energy: 35; collision energy spread: 20. Compounds’ identification was performed using MS-DIAL software version 4.70 and respect library.

### Quality assurance

To ensure the precision of sample pretreatment and the accuracy of the obtained data, the instrument was calibrated and tuned before and after running the sequence of samples using MS calibration kits From AB Sciex^®^. Regarding the blank sample, Milli-Q water was initially treated with the same procedures and during data processing using MS-Dial, the blank data was subtracted from all the measured samples.

### Statistical analysis

We started by curating the dataset for all metabolites. The generated dataset developed based on using the height of the metabolites’ signals. The analysis started by grouping the 53 participants into clusters pretending that their clinical diagnosis is unknown. This showed their ability in providing diagnosis based on the height signals and not the clinical diagnosis.

To this end, we used the principal component analysis (PCA) technique to reduce the dimension of the data, which was done by reducing the number of variables of the data set, while preserving as much information as possible. PCA analysis was performed using the K-means (Everitt and Hothorn, 2011).

To successfully perform PCA analysis, we standardized the data. The standardization was done using the function $\log_{2}\left(1+data\right)$ . This function reduces the scale of the variables while retaining data=0 as it is because $\log_{2}\left(1+0\right)=0$ . For visualization purposes, PCA was carried out together with the Silhouette Visualizer with the Silhouette Coefficient to quantify the goodness of clustering scheme (Hastie et al., 2009).

We used Welch’s *t*-test to compare the means of heights of signals obtained from each of the 427 metabolites of the recruited groups. The cohort consisted of the 3 groups: 27 PD patients (P), 18 reference controls (C), 8 high-risk controls (HC). The three groups were completely independent, and the data was unpaired. Additionally, we observed that there were three significantly unequal variances for each metabolite. These features were exactly the assumptions of the Welch’s *t*-test, the standard test that is usually used to test if two groups have the same mean or not under these conditions (Derrick and White, 2016). The test was performed on python (version 3.9) and applied on all possible combinations from the three groups observed (P, C, and HC), namely, (P, C), (C, HC) and (P, HC). The theory of Welch’s *t*-test also requires that the three data sets be normality distributed. This is a strong assumption that in practical application is impossible to hold completely. However, we used the Kolmogorov-Smirnov (KS) (Massey, 1951), Jarque-Bera (JB) (Bera and Jarque, 1981), and Anderson-Darling (AD) (Anderson and Darling, 1954) goodness of fit tests of normality.

KS rejected all sets to come from normal distribution, JB and AD did not reject the normality of 116 metabolites from group (P), 280 from group (C), and 349 from group (HC), and 377 from group (P), 222 from group (C), and 149 from group (HC), respectively. Moreover, JB and AD were complementary to high extent. In fact, at least one of the tests did not reject 422 (99%) metabolites from group (P), 394 (92%) from group (C), and 399 (93%) from group (HC) to be normally distributed. This demonstrates that the data is reasonably normally distributed even with the small sample sizes we have for each group.

To run the Welch’s *t*-test, we first introduced its theory in terms of our data notation. Here, we give the notations of the pair (P, C), and notations for the pairs (C, HC) and (P, HC) are the same. Let ${\bar{X}}_{P,i}$ be the mean of the signals’ height for each metabolite i across the P samples. Similarly, let ${\bar{X}}_{C,i}$ be the same but for the C samples instead. For each, let $n_{P}$ and $n_{C}$ be the number of samples from P and C samples, respectively. Further, let $S_{P,i}^{2}$ and $S_{C,i}^{2}$ be the variances of the samples from P and C , respectively. Here we index i as i=0,1,…,426 corresponding to the 427 metabolites. We used $∆{\bar{X}}_{i}={\bar{X}}_{P,i}-{\bar{X}}_{C,i}$ as a metric for the difference in the signals’ height for each metabolite i , thus, because of independence, the variance of $∆{\bar{X}}_{i}$ was $S_{∆{\bar{X}}_{i}}^{2}=\frac{S_{P,i}^{2}}{n_{P}}+\frac{S_{C,i}^{2}}{n_{C}}$ that was the denominator of the Welch’s *t*-test statistic $t_{Welch,i}=\frac{∆{\bar{X}}_{i}}{S_{∆{\bar{X}}_{i}}}$ , which approximately had t-distribution with $\nu_{i}\approx \frac{S_{∆{\bar{X}}_{i}}^{4}}{d_{i}}$ degrees of freedom, where $d_{i}=\frac{S_{P,i}^{4}}{{n_{P}}^{2}\left(n_{P}-1\right)}+\frac{S_{C,i}^{4}}{{n_{C}}^{2}\left(n_{C}-1\right)}$ . The Welch’s *t*-test was used to find the significantly different metabolite signals between the two groups when the *p*-value p≤0.05 , the significance level of the test.

### Multiple hypothesis correction

After applying Welch’s *t*-test and going through the hypothesis testing framework, we ended the statistical part by performing multiple hypothesis testing corrections. This step is usually done for ensuring that the results obtaining from multiple hypothesis tests that were carried simultaneously were real significant findings and not obtained by chance. In our study, we tested the significance of 427 metabolites; thus, it was crucial to perform this step. During running the Welch’s test, we chose the significance level (α) = 0.05, but, by the correction tests, we adjusted the α to reduce the number of possible false discoveries. Here, we discuss the three most common treatments: the Bonferroni (Aickin and Gensler, 1996), Holm-Bonferroni (Aickin and Gensler, 1996), and Benjamini–Hochberg Corrections (Benjamini, 2010).

#### Bonferroni Correction

In a series of N (number of metabolites) tests, if the significance level of each test is set to α/N, or equivalently if the null hypothesis of each test is rejected when α/N bounds the corresponding *p*-value. This methodology guarantees that the probability of getting at least one false significant result is less than α. The Bonferroni correction applies to a series of tests that are not necessarily independent. However, when the number of tests is large, the rejection criteria are stringent, and this may lead to accepting many false null hypotheses (Nakagawa, 2004). The significant metabolites are the ones found significant after applying the Bonferroni Correction with α=0.05 and N=427 , that is to reject at significance level $\alpha /N\approx 1.17\times 10^{-4}$ .

#### Holm-Bonferroni correction

The Holm-Bonferroni Correction makes adaptive adjustments to the rejection criterion of each test by first ordering the N *p*-values (Corresponding to the N tests) as $p_{1}\leq p_{2}\leq \cdots \leq p_{N}$ , starting with j=1 , which corresponds to the minimum *p*-value, and reject the $j^{th}$ null hypothesis if $p_{j}\leq \frac{\alpha }{N-\left(j-1\right)}$ , proceeding to the next *p*-value incrementing j by 1, and, finally, checking the inequality. We stopped once the inequality did not hold, identifying this event at step k , and did not reject k , k+1 , ⋯ , N *.* This correction also guarantees that the probability of getting at least one false significant result to be less than α (Giacalone et al., 2018).

#### Benjamini–Hochberg correction

The third correction test we performed was the Benjamini–Hochberg Correction test. Unlike the first two corrections, the Benjamini–Hochberg Correction guarantees that the expected proportion of false discoveries among all discoveries made is less than the significance level α. This expected proportion is also called the false discovery rate. The test works as follows: Sort the *p*-values $p_{1}\leq p_{2}\leq \cdots \leq p_{N}$ , then find the maximum j such that $p_{j}\leq \frac{j}{N}\alpha$ , and reject all tests 1,2,⋯,j . Unlike Bonferroni Correction, Benjamini–Hochberg correction is a way to limit the large number of false positives without severely reducing the power (Benjamini, 2010).

## Results

In this study we had 53 plasma samples: 27 samples were collected from PD patients, 18 from reference controls, and 8 from high-risk controls. We obtained 2,785 (74.5%) retention time-exact mass pairs in each sample profile by LC-MS.

**FIGURE 2:**
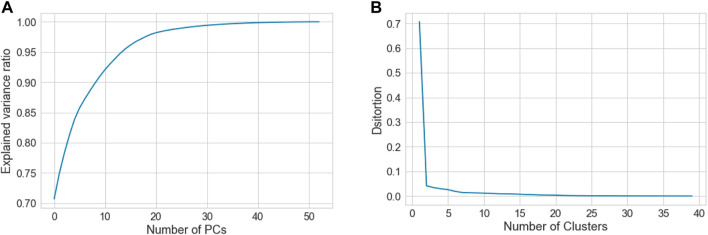
Shows **(A)** graph showing relationship between the number of PCs and the explained variance ratio, and **(B)** Elbow graph, which was used to determine the best clustering scheme for the whole data that consists of the three groups, C, P and H.

To find the suitable number of clusters, we plotted the principal components (PCs) *versus* the explained variance ratio. This showed that two components were sufficient to explain 75% of the variances (Figure 2A), this was a necessary step to run the K-means clustering. On the other hand, we generated K-means elbow graph, which suggested that the best number of clusters should be between 2 and 5, but not more (Figure 2B).

**FIGURE 3:**
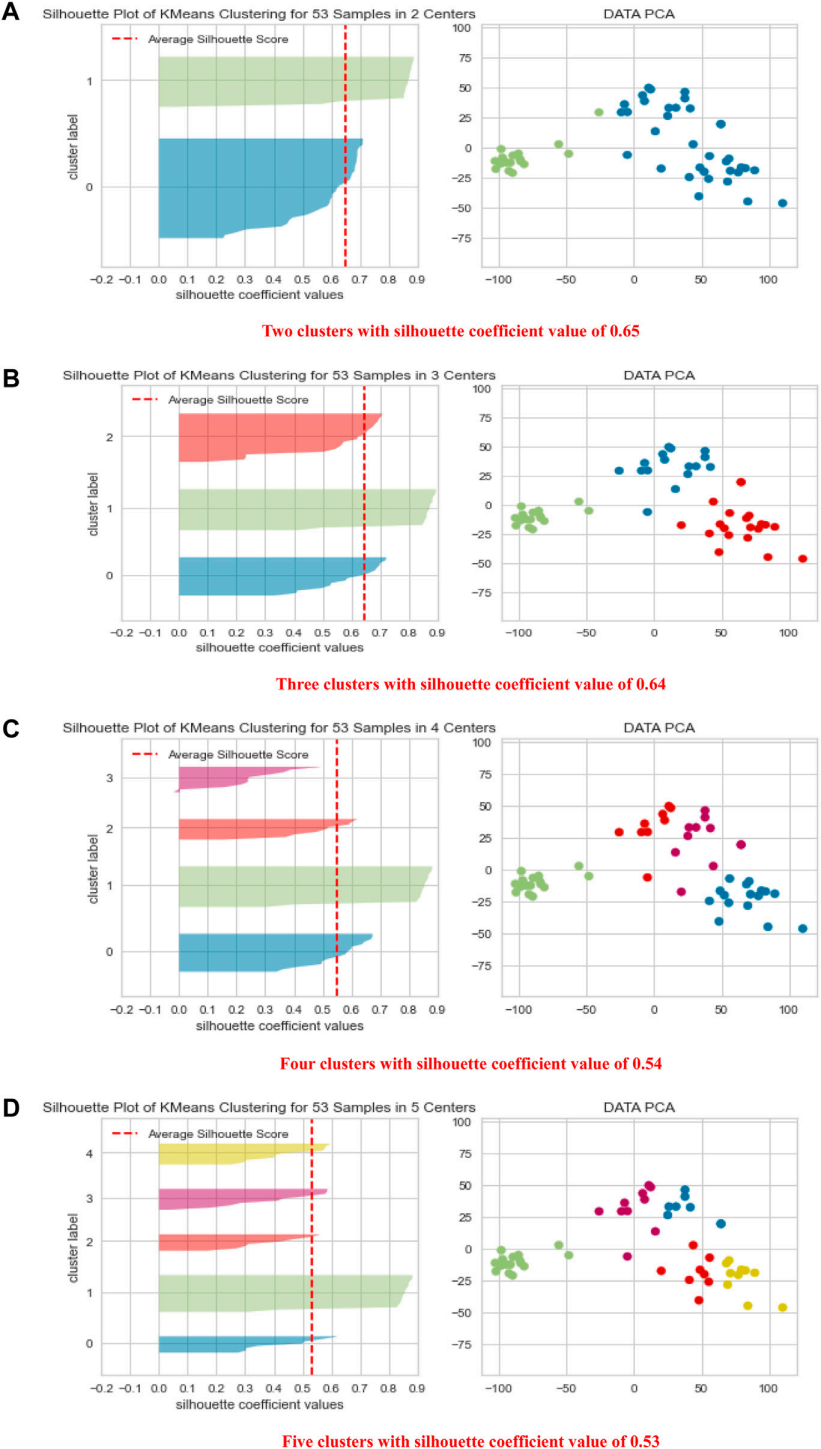
Shows PCA data visualization of 2, 3, 4, and 5 clustering schemes **(A–D)** together with quality clustering indicator, the Silhouette Visualizer with the Silhouette Coefficient.

Figure 3 shows the PCA results for several clustering schemes. The best clustering schemes are of 2 clusters, green (C) and blue (P), or 3 clusters, green (C), red (P), and blue (HC). Both 2-cluster and 3-cluster clustering schemes achieved the highest Silhouette Coefficient. For 4 and 5 clustering system, C is still clearly presented in a separated cluster, which we have colored in green, whereas, P and HC (in different colors) together were further subclustered, showing that they have some subgroups with common features. Figure 3 also demonstrates the high ability of the height of the metabolites’ signals in clustering the groups C, HC and P with high precision.

Interestingly, in the 2-clustering scenario, we found that the agreement between the clustering labels using the k-means algorithm and the clinical diagnoses was 94% in C. This means that the algorithm mistakenly reported only one participant from the C group as P or HC, suggesting that the difference in the levels of metabolites is a powerful tool in clustering the data in high agreement with the clinical diagnosis.

Next, we introduced a comparison of the availability of the 427 metabolites in the three groups, P, H and C using the Welch’s *t*-test. The results of Welch’s test showed that out of 425 detected metabolites, 324 metabolites were found to have significantly different levels in P compared to C (Supplementary Appendix S1). We also found that the majority of the metabolites were significantly higher in C than both P and HC, and few were significantly higher in P or HC than C (summarized in Table 1).

**TABLE 1:** Shows the numbers of significantly different metabolites, which are explained as follows: 313 were significantly higher in C than P, and 11 were significantly higher in P than C. 307 were more significantly available in C than HC, and 4 were significantly higher in HC than C. 4 were significantly higher in HC than P, and 22 were significantly higher in P than HC. Focusing on the metabolic pathways of dopamine, its precursors, and their metabolites, we found significant differences in the levels of these metabolites between C and P (Figure 4) and between C and HC (except for L-tryptophan). However, no significant differences in the levels of the same metabolites between P and HC were identified, except for nicotine (Table 5).

The significantly different metabolites among the 3 groups:P, R and HC		
P	C	HC
	313 P	4 P
11 C		4 C
22 HC	307 HC	

**FIGURE 4:**
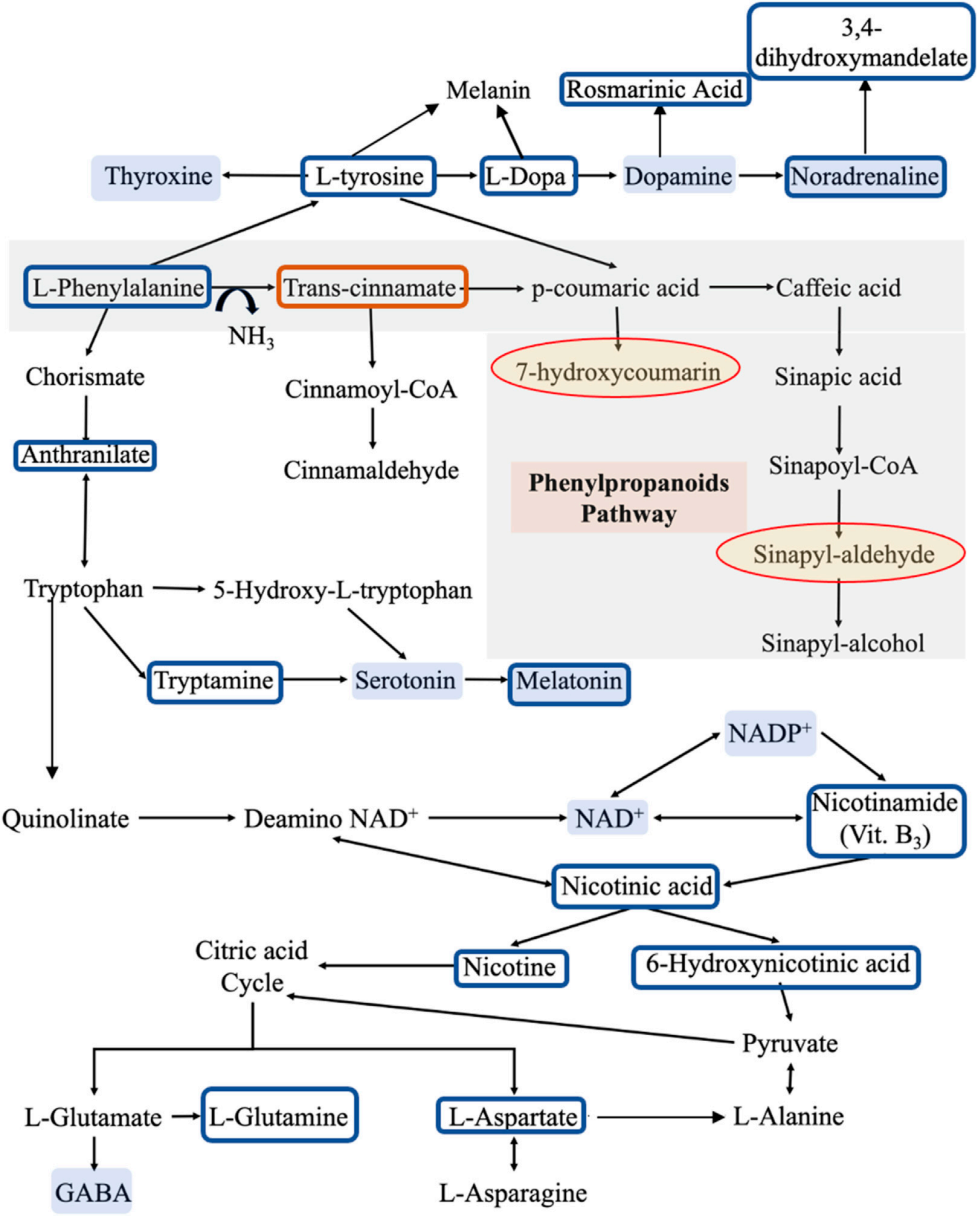
The metabolic pathways for the identified metabolites. Compared to reference controls (C), the blue boxes show the metabolites that are significantly lower in patients (P), while the orange box shows the metabolite that is significantly higher in patients. On the other hand, the yellow shaded circles represent the metabolites that appear in patients only and not in controls, while the blue shaded metabolites are some molecules that may have a role in developing the clinical symptoms of PD according to literature. This pathway was derived from KEGG database.

A complete list of the *p*-values for 324 metabolites significantly different levels among the recruited group can be found in Supplementary Appendix S1 for the comparison of C vs. P. Similarly, Supplementary Appendixs S2, S3 show the complete lists of *p*-values of the 311 and 26 significantly different metabolites for the comparison of C vs. HC, and P vs. HC, respectively. Additionally, we categorized the rest of our findings into 4 groups. Group 1 consists of the metabolites that their levels showed significant alterations among the 3 recruited groups: C, P, and HC, which had been further sub-grouped into 3 clusters as clear from Table 2.

**TABLE 2:** Represents the significant differences in the levels of the tackled metabolites between the different recruited groups: C vs. P, P vs. HC, and C vs. HC. This group is further divided into 3 clusters. Cluster 1 shows metabolites that were higher in C than in HC than in P, while cluster 2 exhibits metabolites that showed no significant differences between C and P but showed significant differences between those 2 latter recruited groups and HC. On the other hand, cluster 3 shows metabolites that have been significantly higher in C than in P and in HC. Noting that (>) means significantly higher and (≈) means not significantly different. Group 2 contains metabolites that were significantly unchanged either between C and HC or between HC and P but were significantly altered between C and P (Figure 5). Group 3 contains metabolites with levels significantly higher in P than in C (Table 6), while group 4 contains metabolites that were significantly presented in HC and/or P only (Table 7).

Cluster number	Intensity level	Metabolite	C vs. P	P vs. HC	C vs. HC
			*p*-values (%)		
1	**C > H > P**	4-Methylsulfinylbutyl glucosinolate	0.57	3.7	3.18
		4-Nonanolide	0.01	4.16	0.03
		gamma-Glu-Cys	0.02	2.69	0.02
		Histidine	0.01	4.65	0.04
2	**C** ≈ **P > H**	2-Methyllactic Acid	20.4	0.42	0.02
		Calciferol	8.4	3.63	1.17
3	**C > P > H**	(+-)-Jasmonic acid	1.4e^−4^	4.45	1.2e^−4^
		1,16-Hexadecanediol	0.01	3.65	0.01
		16-Hydroxyhexadecanoic acid	6.3e^−6^	2.63	5.49e^−6^
		2′-Deoxyinosine 5′-monophosphate	3.13e^−4^	2.22	6.7e^−5^
		beta-Nicotinamide mononucleotide	0.04	2.85	8.12e^−4^
		Creatinine	1.0e^−5^	3.79	5.8e^−6^
		Cytidine	0.04	1.6	0.04
		Daphnetin	0.29	0.8	0.14
		DL-Cystathionine	0.01	0.25	1.9e^−3^
		Histamine	6.5e^−6^	2.55	2.8e^−6^
		N-Palmitoyl-D-erythro-Sphingosine	0.15	3.03	4.7e^−3^
		Nicotine	0.09	2.8	0.03
		Phytol	0.04	4.53	0.01
		Quercetin	0.1	0.27	0.04
		Sorbitol 6-phosphate	0.84	0.52	0.75
		Leupeptin hemisulfate salt	1.78	3.0	0.15
		Sarsasapogenin	2.11	0.62	0.03

**FIGURE 5:**
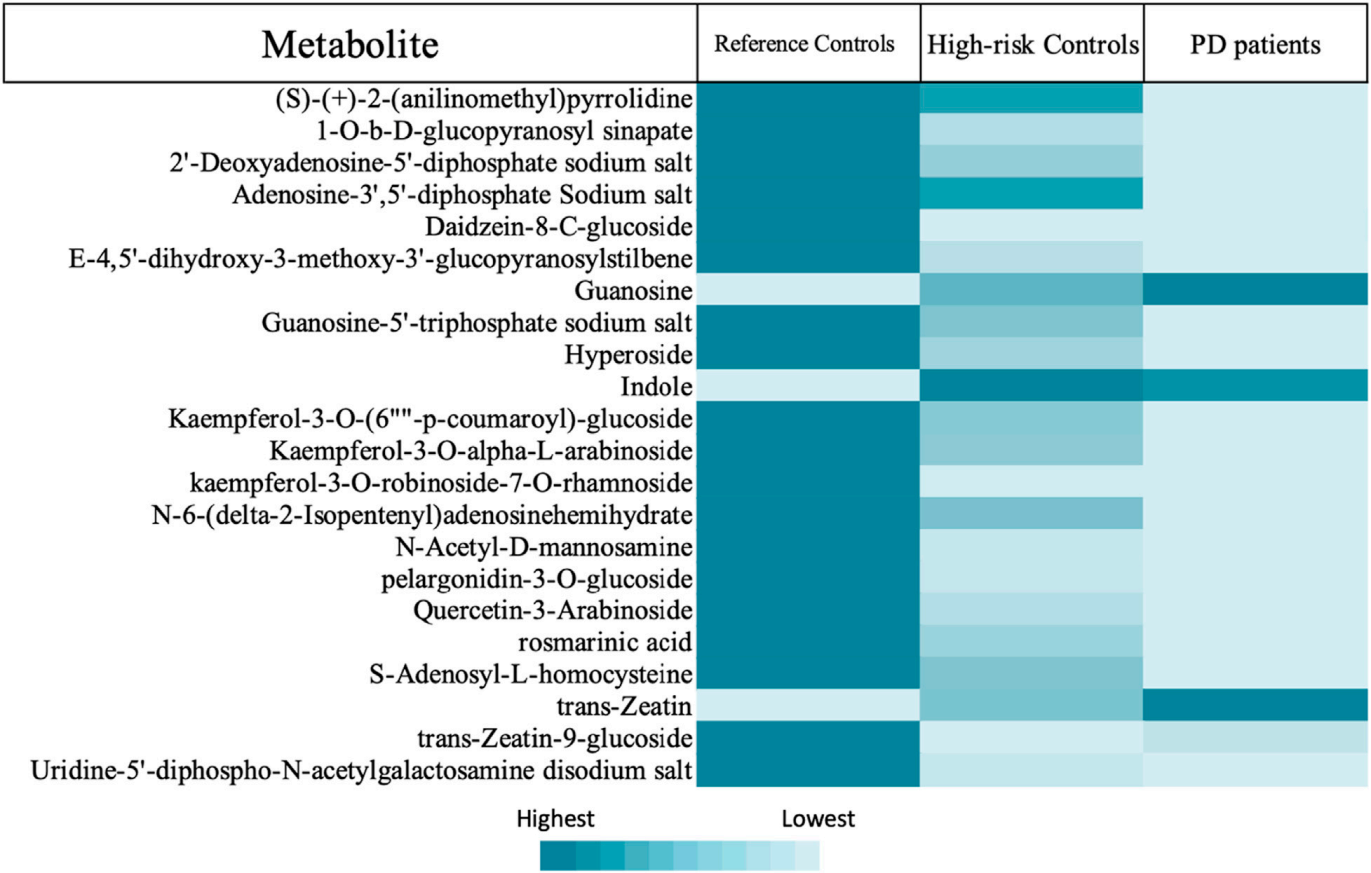
Shows heatmap representing the metabolites of group 2.

### Multiple hypothesis correction

Bonferroni Correction confirmed that the levels of 155 metabolites were altered between C and P groups instead of 324 (Supplementary Appendix S1), 146 instead of 311 metabolites between C vs. HC groups (Supplementary Appendix S2), and zero instead of 26 metabolites between P and HC groups (Supplementary Appendix S3). In contrast, Holm-Bonferroni confirmed that the levels of 168 metabolites were altered between C and P groups instead of 324 (Supplementary Appendix S1), 159 instead of 311 metabolites between C vs. HC groups (Supplementary Appendix S2), and zero instead of 26 metabolites between P and HC groups (Supplementary Appendix S3). On the other hand, Benjamini–Hochberg Correction confirmed that the levels of 321 metabolites were altered between C and P groups instead of 324 (Supplementary Appendix S1), 298 instead of 311 metabolites between C vs. HC groups (Supplementary Appendix S2), and zero instead of 26 metabolites between P and T groups (Supplementary Appendix S3). Tables 3, 4 show the significant difference in the levels, fold changes and direction of the tackled metabolites previously mentioned in Table 5 between P and C after the Correction tests.

**TABLE 3:** Shows whether each of the altered metabolites exhibited significant difference between P and C after running the Correction tests: Bonferroni Correction, Holm-Bonferroni, and Benjamini–Hochberg Correction.

Metabolite	C vs. P *p*-value (%)	Bonferroni correction	Holm-bonferroni	Benjamini–hochberg correction
3,4-Dihydroxymandelate	**0.66**	**Not Sig**	**Not Sig**	**Sig**
3-Hydroxyanthranilic Acid	**7.6e** ^**−**^ **^4^**	**Sig**	**Sig**	**Sig**
Glucose 6-Phosphate	**0.74**	**Not Sig**	**Not Sig**	**Sig**
L-Dopa (3,4-Dihydroxy-L-Phenylalanine)	**8.1e** ^**−**^ **^5^**	**Sig**	**Sig**	**Sig**
L-Phenylalanine	**1.4e** ^**−**^ **^4^**	**Sig**	**Sig**	**Sig**
L-Aspartic Acid	**3.5e** ^**−**^ **^5^**	**Sig**	**Sig**	**Sig**
L-Glutamine	**7.5e** ^**−**^ **^3^**	**Not Sig**	**Not Sig**	**Sig**
L-Tryptophan	**16.16**	**Not Sig**	**Not Sig**	**Not Sig**
Melatonin	**4.4e** ^**−**^ **^5^**	**Sig**	**Sig**	**Sig**
NADH	**0.1**	**Not Sig**	**Not Sig**	**Sig**
Nicotinamide	**0.45**	**Not Sig**	**Not Sig**	**Sig**
Nicotine	**0.09**	**Not Sig**	**Not Sig**	**Sig**
Nicotinic Acid	**5.2e** ^**−**^ **^4^**	**Sig**	**Sig**	**Sig**
Norepinephrine (Noradrenaline)	**0.005**	**Sig**	**Sig**	**Sig**
Pyridoxal 5-Phosphate	**0.23**	**Not Sig**	**Not Sig**	**Sig**
Pyridoxamine (higher in P than in C)	**0.01**	**Not Sig**	**Sig**	**Sig**
Rosmarinic Acid	**0.41**	**Not Sig**	**Not Sig**	**Sig**
Trans-Cinnamate (higher in P and H than in C)	**0.01**	**Sig**	**Sig**	**Sig**
Tryptamine (3-(2-Aminoethyl)Indole)	**2.79**	**Not Sig**	**Not Sig**	**Sig**
Tyrosine	**9.3e** ^**−**^ **^4^**	**Sig**	**Sig**	**Sig**

**TABLE 4:** Shows the fold change and its direction for each significantly altered metabolite between C and P.

Metabolite	C vs. P *p*-value (%)	Fold change	Direction of change
3,4-Dihydroxymandelate	**0.66**	**12.17**	**Higher in C**
3-Hydroxyanthranilic Acid	**7.6e** ^**−**^ **^4^**	**6.44**	**Higher in C**
Glucose 6-Phosphate	**0.74**	**13.9**	**Higher in C**
L-Dopa (3,4-Dihydroxy-L-Phenylalanine)	**8.1e** ^**−**^ **^5^**	**165.33**	**Higher in C**
L-Phenylalanine	**1.4e** ^**−**^ **^4^**	**9.38**	**Higher in C**
L-Aspartic Acid	**3.5e** ^**−**^ **^5^**	**6.28**	**Higher in C**
L-Glutamine	**7.5e** ^**−**^ **^3^**	**25.8**	**Higher in C**
L-Tryptophan	**16.16**	**Not Sig**	**Not Sig**
Melatonin	**4.4e** ^**−**^ **^5^**	**8.63**	**Higher in C**
NADH	**0.1**	**7.08**	**Higher in C**
Nicotinamide	**0.45**	**2.04**	**Higher in C**
Nicotine	**0.09**	**3.37**	**Higher in C**
Nicotinic Acid	**5.2e** ^**−**^ **^4^**	**7.83**	**Higher in C**
Norepinephrine (Noradrenaline)	**0.005**	**6.93**	**Higher in C**
Pyridoxal 5-Phosphate	**0.23**	**43.08**	**Higher in C**
Pyridoxamine (higher in P than in C)	**0.01**	**1.63**	**Higher in** **P**
Rosmarinic Acid	**0.41**	**2.26**	**Higher in C**
Trans-Cinnamate (higher in P and H than in C)	**0.01**	**1.68**	**Higher in** **P**
Tryptamine (3-(2-Aminoethyl)Indole)	**2.79**	**7.61**	**Higher in C**
Tyrosine	**9.3e** ^**−**^ **^4^**	**10.93**	**Higher in C**

**TABLE 5:** Represents the significant differences (*p*-value%) in the levels of the tackled metabolites between the different recruited groups: C vs. P, P vs. HC, and C vs. HC. The yellow shaded cells shows insignificant difference, while the orange cells represents metabolites that were significantly higher in P than in C; however, the rest of the metabolites were significantly higher in C than in P.

Metabolite	C vs. P *p*-value (%)	P vs. HC *p*-value (%)	C vs. HC *p*-value (%)
3,4-Dihydroxymandelate	**0.66**	**56.76**	**0.73**
3-Hydroxyanthranilic Acid	**7.6e** ^**−**^ **^4^**	**93.21**	**7.1e** ^**−**^ **^4^**
Glucose 6-Phosphate	**0.74**	**94.17**	**0.76**
L-Dopa (3,4-Dihydroxy-L-Phenylalanine)	**8.1e** ^**−**^ **^5^**	**46.23**	**7.9e** ^**−**^ **^5^**
L-Phenylalanine	**1.4e** ^**−**^ **^4^**	**16.63**	**1.0e** ^**−**^ **^4^**
L-Aspartic Acid	**3.5e** ^**−**^ **^5^**	**36.52**	**1.7e** ^**−**^ **^5^**
L-Glutamine	**7.5e** ^**−**^ **^3^**	**7.0**	**0.01**
L-Tryptophan	**16.16**	**39.3**	**16.75**
Melatonin	**4.4e** ^**−**^ **^5^**	**33.12**	**0.23**
NADH	**0.1**	**82.45**	**0.21**
Nicotinamide	**0.45**	**75.2**	**1.19**
Nicotine	**0.09**	**2.8**	**0.03**
Nicotinic Acid	**5.2e** ^**−**^ **^4^**	**91.47**	**4.3e** ^**−**^ **^4^**
Norepinephrine (Noradrenaline)	**0.005**	**79.59**	**0.01**
Pyridoxal 5-Phosphate	**0.23**	**73.6**	**0.23**
Pyridoxamine (higher in P than in C)	**0.01**	**6.54**	**12**
Rosmarinic Acid	**0.41**	**40.03**	**6.86**
Trans-Cinnamate (higher in P and H than in C)	**0.01**	**57.99**	**3.56**
Tryptamine (3-(2-Aminoethyl)Indole)	**2.79**	**64.21**	**2.43**
Tyrosine	**9.3e** ^**−**^ **^4^**	**24.32**	**4.7e** ^**−**^ **^4^**

**TABLE 6:** Represents the metabolites of group 3, which were significantly higher in P than in C.

Metabolite	C vs. P *p*-value (%)
Guanosine	**2.13**
INDOLE	**2.42**
L-beta-Homoproline	**0.99**
L-beta-homotryptophan-HCl	**1.1e** ^**−**^ **^3^**
Pyridoxamine	**0.01**
trans-Cinnamate	**0.01**
trans-Zeatin	**2.25**

**TABLE 7:** Represents the metabolites of group 4, which were significantly available in H and/or in P.

Metabolite	P	HC
Anserine	**Only available in Patients**	
7-Hydroxy-4-Methylcoumarin		
Sinapyl aldehyde	**Available in both patients and high-risk controls**	
GAMMA-TERPINENE		

## Discussion

In this study, we employed LC-MS for comprehensive metabolomic profiling of metabolites in the plasma of 27 idiopathic PD patients, 18 reference controls, and 8 high-risk controls. Statistical analysis was performed using multivariate and univariate analysis to assess the significant differences between the recruited groups. Multivariate analysis (PCA) perfectly stratified the recruited groups in the 2-cluster system into reference controls and PD patients/high-risk controls. Interestingly, the 3-cluster system significantly discriminated between reference controls and PD patients/high-risk controls, and, to a lesser extent, between PD patients and high-risk controls. These results prove that high-risk controls are significantly related to PD patients and suggest that high-risk controls might represent an early stage across the disease spectrum. The univariate analysis showed that out of the screened 427 metabolites, 324 metabolites had significantly different levels in the PD patients compared to the reference controls. Moreover, when we carried out the 3 correction tests, the conclusion was to accept the preliminary generated results from Welch’s test as Benjamini–Hochberg Correction almost confirmed all of them. Although Benjamini–Hochberg Correction accepted the null hypothesis regarding the altered metabolites between P and HC, we also decided to accept the results from Welch’s test due to the small sample size we used.

We focused on phenylalanine metabolism through PAH (tyrosine, DA, and norepinephrine synthesis pathway), PAL (phenylpropanoid pathway), tryptophan, and citrate cycle metabolic pathways. Our findings support that there is a metabolic shift in the phenylalanine metabolism in PD from tyrosine production into producing trans-cinnamate instead. This alteration may deprive the body of synthesizing dopamine, norepinephrine and every other phenylalanine and tyrosine metabolite. Additionally, it could influence every metabolic pathway involving one of the altered metabolites. These findings were confirmed by the several statistical tests we performed, Welch’s *t*-test and the multiple hypothesis correction.

### Trans-cinnamate and phenylalanine metabolism

Significant lower intensities of phenylalanine, tyrosine, L-dopa, rosmarinic acid (dopamine metabolite), norepinephrine, 3,4-Dihydroxymandelate (norepinephrine metabolite) and 3-hydroxyanthranilic acid were detected in P compared to C. On the other hand, trans-cinnamate levels were higher in P than in C. Given that phenylalanine is an essential amino acid (Kohlmeier, 2003), and its conversion into tyrosine is an irreversible reaction (Kaufman, 1999), our findings suggest a shift in the metabolic reaction of phenylalanine via PAL to produce trans-cinnamate instead of being metabolized via PAH and producing tyrosine and, eventually, dopamine and noradrenaline.

This shift in the metabolic pathway could deprive the neurons of dopamine and its metabolites like 3,4-dihydroxyphenylacetaldehyde (DOPAL). A very recent study was done to test the effect of DOPAL on α-syn-induced neurotoxicity, and they found that DOPAL showed a significant effect on preventing α-syn aggregation and induced neurotoxicity (Gallardo-Fernández et al., 2023). Moreover, this shift in the phenylalanine metabolic pathway produces higher amounts of ammonia which has been proven to be neurotoxic agent (Oliphant and Allen-Vercoe, 2019; Chang and Chen, 2020). Several studies have reported lower levels of phenylalanine and its metabolite, beta-phenylethylamine, in PD patients than in controls (Braham et al., 1969; Zhou et al., 1997; MIURA, 2000). One of these studies measured the levels of phenylalanine and tyrosine after oral phenylalanine and tyrosine tolerance test, where PD patients exhibited lower levels of phenylalanine while having normal tyrosine levels. This was not related to malabsorption of phenylalanine (Braham et al., 1969). Our findings may support this. Although phenylalanine and tryptophan are essential amino acids, only phenylalanine showed to have significantly lower levels in P than in C, while there were no significant differences in the levels of tryptophan between the recruited groups. Additionally, another study found that the tyrosine/phenylalanine ratio in PD patients is lower than that in controls (Hirayama et al., 2016), this suggests that the metabolic pathway of phenylalanine (through PAH) in PD may shift to another one that does not end by tyrosine. Indeed, recently PD patients showed lower activity in their PAH enzyme (Steventon and Mitchell, 2018; Rawlings et al., 2019). All these studies support our hypothesis that the metabolism of phenylalanine in PD patients is altered via the following mechanisms: 1) reduction of PAH activity, which results in metabolizing phenylalanine through the PAL metabolic pathways, thus, reducing the production of tyrosine, and, subsequently, dopamine and norepinephrine; 2) enhancing PAL activity with the resultant increase in the production of trans-cinnamate and ammonia.

**FIGURE 6:**
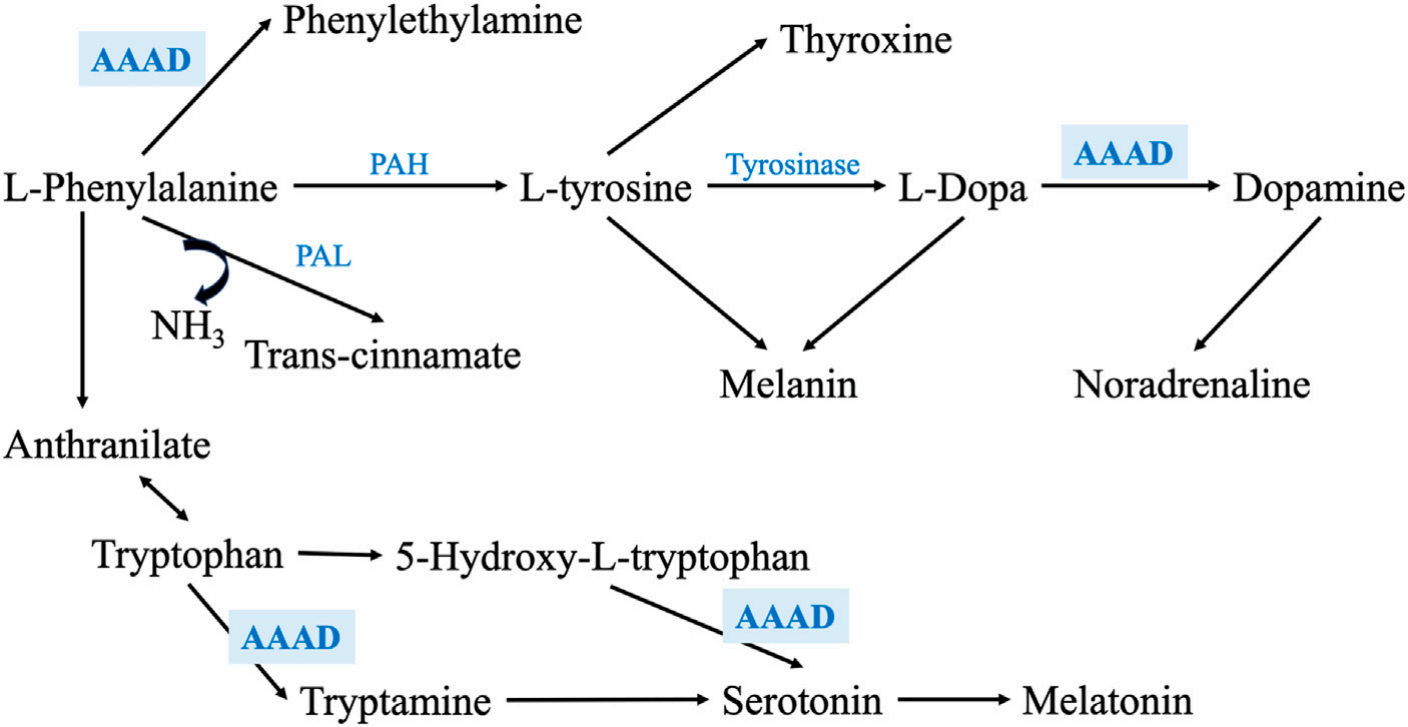
Shows a zoom-in into the phenylalanine metabolism through PAH and PAL enzymes, exhibiting the main metabolic enzymes involved in this pathway. It also illustrates how AAAD is involved not only phenylalanine and L-Dopa metabolism, but also in tryptophan metabolism and serotonin synthesis.

On the other hand, AAAD is involved in the metabolism of several of the assayed metabolites. For example, it metabolizes phenylalanine into phenylethylamine, L-Dopa into DA, tryptophan into tryptamine, and 5-hydroxytryptophan into serotonin (Figure 6) (Zhu et al., 1992; Gücüyener et al., 2014); thus, any alteration in this enzyme’s levels may impact the metabolism of these metabolites. As the regulation of any enzyme is affected by the concentration and the availability of its substrate (Aragón and Sols, 1991), the expression and/or activity of AAAD may be altered in PD due to the deficiency of phenylalanine and L-Dopa, which, may affect the metabolism of tryptophan and the synthesis of serotonin and, its metabolites such as melatonin. Moreover, pyridoxamine and pyridoxal 5′-phosphate (PLP) are 2 interchangeable forms of vitamin B_6_ in the human body (Oppici et al., 2015). PLP, the active form of vitamin B6, is a cofactor for several B6-dependent (PLP-dependent) enzymes involved in many vital cellular processes. One of these enzymes is AAAD (Al Mughram et al., 2022).

Since our PD cohort is on treatment (mixture of L-Dopa/Carbidopa), they were expected to have lower levels of PLP as it is the binding target for Carbidopa (Bertoldi, 2014). Our results showed that the levels of pyridoxamine were significantly higher in PD patients than in reference control. In comparison, the levels of PLP were significantly lower in PD patients than in reference controls. These findings may suggest that the conversion, itself, of the inactive form of vit. B6, pyridoxamine, to the active form, PLP, is lower in PD patients than in reference controls, which may further decrease the activity of AAAD and impact its downstream metabolic pathway in the patients. Our findings showed that there were no significant difference in the levels of pyridoxamine between HC and P or between HC and C, which may suggest that the conversion reaction from the inactive to the active form is affected by the transformation from being C to HC and, thus, P. Interestingly, PLP is a cofactor for 300 enzymes, including mitochondrial enzymes and many other enzymes involved in the metabolism of several metabolites of our targeted metabolic pathway (*Pyridoxal in Homo Sapiens in UniProtKB Search* (*328*) UniProt, 2023).

Moreover, the appearance of some phenylpropanoids’ metabolites such as 7-hydroxy-4-methylcoumarin (methylated metabolite of 7-hydroxycoumarin) and Sinapyl-aldehyde in P only and not in C may support the switch in metabolic pathways of phenylalanine in PD patients.

A study done on 92 idiopathic PD patients and 65 normal controls, detected coumaric acid, the precursor of 7-hydroxycoumarin, in PD patients, which confirms our findings (Shao et al., 2021). Recently, it was shown that synthetic 7-hydroxy-4-methylcoumarin stimulates tyrosinase, an enzyme that converts tyrosine to L-dopa (Kim et al., 2023). We believe that this metabolite appeared in patients due to the shifting of the reaction towards trans-cinnamate instead of tyrosine, with the resulting positive biochemical feedback mechanisms on tyrosinase in an attempt to activate the tyrosinase and produce L-dopa in patients (Cinquin and Demongeot, 2002; Kleppe et al., 2021).

In addition, we detected significantly lower levels of 3-hydroxyanthranilic acid, a metabolite of anthranilate, in PD patients than in reference controls. As annotated by the KEGG database, anthranilate is a metabolite that links phenylalanine to tryptophan metabolism. This finding was confirmed by previous study that found a significant alteration of the same metabolite in PD patients compared to controls (Shao et al., 2021).

### Tyrosine metabolism

As described in the previous section, the shift in phenylalanine metabolism, decreasing tyrosine level will lower its metabolites, namely, L-dopa, DA, norepinephrine, thyroxine, and melanin (Rzepka et al., 2016; Dratman and Martin, 2020; Genome, 2023c; Lopez and Mohiuddin, 2023).

We detected lower levels of dopamine and norepinephrine in P than in C, confirming that the tyrosine levels were deficient due to the reduction in phenylalanine metabolism into tyrosine. Furthermore, we found that norepinephrine levels were lower in P than in C which is aligned to previous research (Eldrup et al., 2009; Delaville et al., 2011). It is suggested that the reduced levels of norepinephrine in PD may be responsible for various features of autonomic impairments. According to Braak’s theory, an initial loss of the adrenergic neurons occurs before the degeneration of the dopaminergic ones (Espay et al., 2014). However, our findings may suggest that the autonomic signs may have appeared in PD’s prodromal phase as an initial sign of the phenylalanine metabolic disorder that occurs in PD patients, producing lower levels of norepinephrine.

Tyrosine is also the precursor of Thyroid hormone which plays a vital role in regulating cellular metabolism, modulating neurotransmission and supporting neurodevelopment (Charoenngam et al., 2022). The DA-thyroxine relationship is very complicated. In addition to sharing the same precursor, dopamine has a regulatory role in thyroxine expression. Dopamine upregulates the expression of TRH (thyrotropin-releasing hormone) and downregulates TSH (thyroxine-stimulating hormone) (Mohammadi et al., 2021). Changes in the levels of thyroid hormone is reflected on modulating the dopaminergic receptors and their sensitivity (Crocker and Cameron, 1988). A recent meta-analysis study, revealed that there is a significant correlation between thyroid dysfunction, hypothyroidism and hyperthyroidism, and the risk of developing PD (Charoenngam et al., 2022). Based on our findings supporting the shift in phenylalanine metabolism towards trans-cinnamate production instead of tyrosine in PD, we assume that the lower levels of tyrosine in P lead to the production of lower levels of thyroxine, resulting in developing hypothyroidism. Since we had excluded any participant with a history of thyroid dysfunction disorder or on thyroid hormone replacement therapy and given that thyroxine regulates glucose metabolism (Mendez and Ortiz, 2021), we assessed the levels of glucose-6-phosphate between P and C. Our results showed that P have significantly lower levels of this metabolite, confirming our hypothesis that the shift in phenylalanine metabolism that led to a decrease in the levels of tyrosine has an impact on thyroxine level and its physiological functions as a consequence. This finding may also explain the fatigue that most PD patients have reported (Lin et al., 2021).

Tyrosine and L-DOPA are considered bioregulators for melanogenesis and other cellular functions (Slominski et al., 2012) which are proven to be affected in PD patients who suffer from selective neurodegeneration of neuromelanin-containing neurons, particularly, the substantia nigra dopaminergic neurons (Carballo-Carbajal et al., 2019). Additionally, several studies reported that individuals with lighter pigmentation or cutaneous malignant melanoma have a significantly higher incidence of PD (Krainc et al., 2023).

### Tryptophan metabolism

Although we did not find significant differences in the levels of tryptophan between PD patients and reference controls, our results showed significantly lower levels of tryptamine (3-(2-aminoethyl) indole), which is one of the primary metabolites of tryptophan and the precursor of both serotonin and melatonin, in PD patients (Genome, 2023b). This may confirm that the absorption of amino acids such as tryptophan is normal, unlike its metabolism.

In the present study, melatonin levels were significantly lower in P compared to controls, which may be linked to sleep disorders in PD. Several previous studies support our findings. For instance, one study done by scientists from Cambridge University on 12 PD patients and 12 controls found that melatonin levels were not only deficient in PD patients compared to controls but also accompanied by hypothalamic volume loss (Breen et al., 2016). In, another study that was done on 20 PD patients and 15 controls to measure the melatonin levels at 30-min intervals for 24 h and correlate these measurements to the sleep quality (Pittsburgh Sleep Quality Index) and daytime sleepiness (Epworth Sleepiness Scale), found a significant reduction in the melatonin levels in PD patients compared to controls (Videnovic et al., 2014).

Although we did not assess the levels of quinolinate, we assessed the levels of NADH, another form of NAD + which is a tryptophan metabolite. We found lower levels of NADH, nicotinamide, nicotinic acid (quinolinate metabolites, and the precursors and metabolites of NAD+ and NADP+) in P than in C. This reduction in NAD levels may have a role in the neurotoxicity in PD. These findings are aligned with previous research; for example, one study found that NAD levels were significantly lower in the muscles of 30 PD patients compared to age-matched controls (Mischley et al., 2023), while a clinical trial phase I study tested the effect of oral administration of nicotinamide riboside (NR) in 30 PD patients, and found that NR increases the levels of NAD, stimulates the transcriptional upregulation of the processes related to mitochondrial, lysosomal, and proteasomal activities in skeletal muscles and blood cells, and decrease the inflammatory cytokines (Brakedal et al., 2022). Another study found that NR increased NAD, which led to not only ameliorating the mitochondrial functions in induced pluripotent stem cells isolated from PD patients but also preventing age-related dopaminergic neuronal loss and motor deficits in fly models of GBA-PD (Schöndorf et al., 2018).

The previous findings were also reflected on significantly lower levels of nicotine and 6-hydroxynicotinic acid, nicotinic acid metabolites, in P than in C. Since these latter metabolites are substrates of citrate cycle, we investigated the levels of citrate cycle’s non-essential amino acids metabolites such as L-glutamine and L-aspartic acid. We found that these amino acids were lower in P than in C, aligning with MCA theory and our hypothesis that all the discussed metabolites are acting in a connected network, representing PD as a complex metabolic disorder that could be triggered by the initial shift in the phenylalanine metabolism into trans-cinnamate instead of tyrosine.

### Categorization of metabolites

We categorized some of the detected metabolites into 4 groups. The first group had been subcategorized into 3 clusters. The first cluster contains metabolites that were significantly decreasing in the order C > HC > P. In this cluster, the high-risk group significantly acts as a transition state between the C and P. The second cluster consists of metabolites that showed no significant difference between C and P; however, both groups showed significantly higher levels of these metabolites than HC. Cluster 2 contains only 2 metabolites, which are 2-methyl lactic acid and calciferol. 2-Methyl lactic acid is a methylated metabolite of lactic acid. Finally, the third cluster in group 1 contains metabolites that were significantly higher in C than in both P and HC, and they were significantly lower in HC than in P.

Group 2 is composed of metabolites that showed no significant differences either between C and HC or between HC and P; however, there is a significant difference between P and C. In this group of metabolites, the HC seems to look like a transition between C and P, especially since one of these metabolites is rosmarinic acid, a dopamine metabolite. The levels of rosmarinic acid showed a gradual decrease from C, HC, to P. Moreover, guanosine, another metabolite in group 2, found to be gradually elevated in order C < HC < P; conversely, its metabolite, guanosine 5′-triphosphate sodium salt (GTP metabolite) was gradually decreasing in the opposite order, which means that guanosine was metabolized into GTP in C higher than in HC (not significantly) than in P (significantly compared to reference controls).

Group 3 shows metabolites that were significantly higher in P than in C, including trans-cinnamate, pyridoxamine, and guanosine. In addition, this group also included indole, which is one of tryptophan metabolites (Genome, 2023b). Finally, group 4 contains two metabolites, 7-hydroxy-4-methylcoumarin and anserine, that appeared only in P. Anserine is a dipeptide containing beta-alanine and 3-methylhistidine, a more metabolically stable derivative of carnosine, which is a protein building block that is found in high concentrations in the brain, muscles, and gastrointestinal tissues of humans and all vertebrates (Kwiatkowski et al., 2018; Jukić et al., 2021); this may justify why P showed significantly lower levels of carnosine, beta-alanine, and histidine than reference controls did. Several studies found that the administration of carnosine and anserine provides neuroprotection against neuronal injury (Kaneko et al., 2017). Additionally, group 4 includes 2 metabolites that appeared in both P and HC only and not in C. One of them is gamma-terpinene, a lipid secondary metabolite that is usually produced as a defensive or signaling molecule, and in some cases, they are produced due to incomplete metabolism (Silva et al., 2012; Ahmed et al., 2013; Raman et al., 2013). Moreover, there is a number of studies reported altered lipid metabolism in PD (Alecu and Bennett, 2019). Some studies found that gamma-terpinene possesses anti-inflammatory activity and proved that it can modulate acute inflammatory response in mice (Ramalho et al., 2015). The second metabolite that was available in both P and HC but not in C is sinapyl aldehyde. It is another metabolite from the same phenylalanine metabolism via the phenylpropanoid pathway.

## Conclusion

In our study, we have introduced the switch in phenylalanine metabolism as potential contributor to PD pathogenesis. Based on our findings, we hypothesize that the switch between PAH to PAL phenylalanine metabolic pathways, produces higher amounts of trans-cinnamate instead of the proper amount of tyrosine. As a result, there is a severe decrease in the production of dopamine and significant alterations in the metabolism of several interconnected metabolites such as norepinephrine, thyroxine, and melanin. Being connected to phenylalanine metabolism, tryptophan and citrate cycle metabolism have also been affected. These alterations were reflected on the levels of their metabolites such as serotonin, melatonin, nicotinamide (NAD precursor), and some non-essential amino acids such as L-alanine, L-glutamine (L-glutamate and GABA precursor), and L-aspartate. Based on the literature, most of these altered metabolites have been associated with several signs and symptoms of PD. Thus, we assume that this metabolic shift may be the initiator of the dopaminergic, adrenergic, and serotonergic neurodegeneration in PD as a complex metabolic disorder.

## Data Availability

The original contributions presented in the study are publicly available. This data can be found here: https://osf.io/hdjub/?view_only=8ca85a03a4d74499971d0cb673f202d3.
