# Supplementary material for: Metabolomic profiling reveals altered phenylalanine metabolism in Parkinson’s disease in an Egyptian cohort
Source: Front Mol Biosci. 2024 Mar 7;11:1341950. doi: 10.3389/fmolb.2024.1341950 (PMC10955577; doi:10.3389/fmolb.2024.1341950)
Supplement: Supplementary file 1 [file Table1.DOCX]

**Appendix 1: The p-values of the Welch's t-test when comparing C vs P**

| **Metabolites** | **P-Values** |
| --- | --- |
| (-)-Alpha-Copaene | 0.68% |
| (-)-Epicatechin | 0.00% |
| (-)-Riboflavin | 0.00% |
| (+-)-Alpha-Tocopherol Acetateacid Ester | 0.01% |
| (+-)-Jasmonic Acid | 0.00% |
| (+/-)-Cis,Trans-Abscisic Acid | 0.08% |
| (S)-(+)-2-(Anilinomethyl)Pyrrolidine | 0.08% |
| 1-Aminocyclopropane-1-Carboxylate | 0.00% |
| 1-Hexylamine | 0.00% |
| 1-Methylhistamine | 0.01% |
| 1-Myristoyl-2-Hydroxy-Sn-Glycero-3-Phosphate | 0.74% |
| 1-Myristoyl-2-Hydroxy-Sn-Glycero-3-Phosphoethanolamine | 0.41% |
| 1-O-B-D-Glucopyranosyl Sinapate | 2.02% |
| 1,16-Hexadecanediol | 0.01% |
| 1,2-Dilauroyl-Sn-Glycero-3-Phosphate | 1.09% |
| 1,2-Dipalmitoyl-Sn-Glycero-3-Phospho-Rac-(1-Glycerol) Sodium Salt | 0.04% |
| 1,3-Dimethylurate | 0.00% |
| 1,4-Benzoquinone | 0.00% |
| 16-Hydroxyhexadecanoic Acid | 0.00% |
| 1h-Indole-3-Carboxylic Acid | 0.13% |
| 2-(4-Isobutylphenyl)Propionic Acid | 0.12% |
| 2-Mercaptoethanesulfonic Acid | 0.00% |
| 2,3-Diaminopropionate | 0.00% |
| 2,3-Pyridinedicarboxylic Acid | 0.03% |
| 2,5-Dihydroxybenzoic Acid | 0.03% |
| 2'-Deoxyadenosine | 0.00% |
| 2'-Deoxyadenosine 5'-Monophosphate | 0.00% |
| 2'-Deoxyadenosine-5'-Diphosphate Sodium Salt | 1.59% |
| 2'-Deoxycytidine | 0.00% |
| 2'-Deoxyguanosine 5'-Monophosphate | 0.78% |
| 2'-Deoxyinosine | 0.00% |
| 2'-Deoxyinosine 5'-Monophosphate | 0.00% |
| 2'-Deoxyuridine | 0.35% |
| 2'-Deoxyuridine-5'-Monophosphate | 0.00% |
| 3 5 7-Trihydroxy-4'-Methoxyflavone | 0.00% |
| 3-(2-Aminoethyl)Indole | 2.79% |
| 3-(4-Hydroxy-3-Methoxyphenyl)Prop-2-Enoicacid | 0.00% |
| 3-(4-Hydroxy-3,5-Dimethoxyphenyl)-2-Propenoic Acid | 0.01% |
| 3-(4-Hydroxyphenyl)Propionic Acid | 0.02% |
| 3-(Methylsulfinyl)Propylglucosinolate | 0.03% |
| 3-Chloro-L-Tyrosine | 0.21% |
| 3-Formylindole | 0.00% |
| 3-Hydroxy-3-Methylglutaric Acid | 0.04% |
| 3-Hydroxyanthranilic Acid | 0.00% |
| 3-Hydroxyisovaleric Acid | 0.00% |
| 3-Methylxanthine | 0.02% |
| 3-Phospho-D-Glycerate | 0.08% |
| 3,4-Dihydroxy-L-Phenylalanine | 0.00% |
| 3,4-Dihydroxymandelate | 0.66% |
| 3,4-Dimethoxycinnamic Acid | 0.20% |
| 3' 4' 5 7-Tetrahydroxyflavanone | 0.00% |
| 3'-Dephosphocoenzyme A | 0.23% |
| 3'-Methoxy-4',5,7-Trihydroxyflavonol | 0.00% |
| 4-(2 Aminoethyl)-Phenol | 0.00% |
| 4-Aminophenol | 0.00% |
| 4-Hydroxy-3-Methoxycinnamaldehyde | 0.00% |
| 4-Hydroxyphenylpyruvic Acid | 0.00% |
| 4-Methyl-5-Thiazoleethanol | 0.00% |
| 4-Methylsulfinylbutyl Glucosinolate | 0.57% |
| 4-Nonanolide | 0.01% |
| 4-Pyridoxate | 0.00% |
| 5-Aminoimidazole-4-Carboxamide-1-Ribofuranosyl 5'-Monophosphate | 0.13% |
| 5-Butylpyridine-2-Carboxylic Acid | 0.01% |
| 6-Hydroxynicotinic Acid | 0.00% |
| 7-Acetoxy-4-Methylcoumarin | 3.22% |
| A-Lipoamide | 3.62% |
| Acacetin | 0.84% |
| Adenine | 0.00% |
| Adenosine | 0.39% |
| Adenosine 3':5'-Cyclicmonophosphate | 0.00% |
| Adenosine 5'-Diphospho-Glucose | 0.22% |
| Agmatine | 0.00% |
| All-Trans-Retinoic Acid | 0.00% |
| Allantoin | 0.00% |
| Alpha-D-Glucose-1,6-Diphosphate | 0.01% |
| Alpha-L-(-)-Fucose 1-Phosphate Bis(Cyclohexylammonium) Salt | 0.03% |
| Alpha-Methyl-Dl-Histidine | 0.00% |
| Alpha-Tocotrienol | 0.11% |
| Amantadine | 0.00% |
| Baclofen | 0.04% |
| Baicalein-7-O-Glucuronide | 0.06% |
| Benzaldehyde | 0.00% |
| Benzamidine | 0.08% |
| Beta-Nicotinamide Mononucleotide | 0.04% |
| Cadaverine | 0.00% |
| Caffeine | 0.04% |
| Camphene | 0.00% |
| Canavanine | 0.00% |
| Canthaxanthin | 0.24% |
| Capsaicin | 0.02% |
| Carnosine | 0.00% |
| Cerulenin | 0.00% |
| Chalcone | 0.00% |
| Chlorogenic Acid | 0.39% |
| Cholecalciferol | 0.02% |
| Choline | 0.00% |
| Cis-Aconitate | 0.10% |
| Cis,Cis-Muconic Acid | 0.00% |
| Citraconic Acid | 0.00% |
| Citramalate | 0.00% |
| Citrulline | 0.00% |
| Creatinine | 0.00% |
| Crotonoyl Coenzyme A Lithium Salt | 1.66% |
| Cyanidin-3-O-(6''-O-(E-P-Coum)-2''-O-(Beta-Xylopyranosyl)-Beta-Glucopyranoside)-5-O-Beta-Glucopyranoside | 0.20% |
| Cyanidin-3-O-Galactoside | 0.51% |
| Cysteine S-Sulfate | 0.07% |
| Cytidine | 0.04% |
| Cytidine 5'-Diphosphocholine | 0.25% |
| Cytidine-3',5'-Cyclicmonophosphate | 0.01% |
| Cytidine-5'-Monophosphate | 0.02% |
| Cytosine | 0.00% |
| D-(+)-Maltose | 0.02% |
| D-Carnitine | 0.00% |
| D-Cysteine | 0.17% |
| D-Ribose 5-Phosphate | 0.00% |
| D,L-Sulforaphane | 0.15% |
| Daidzein | 0.00% |
| Daidzein-8-C-Glucoside | 0.95% |
| Daphnetin | 0.29% |
| Delta-Tocopherol | 0.67% |
| Delta-Tocotrienol | 0.59% |
| Diethanolamine | 0.00% |
| Dihydrocapsaicin | 0.00% |
| Dihydrosphingosine | 0.00% |
| Dihydrouracil | 0.00% |
| Diosmin | 3.05% |
| Dl-5-Hydroxylysine | 1.27% |
| Dl-Alpha,Epsilon-Diaminopimelic Acid | 0.00% |
| Dl-Cystathionine | 0.01% |
| Dl-Dihydrozeatin | 0.01% |
| Dl-Threo-Beta-Methylaspartic Acid | 0.00% |
| Dudp | 0.02% |
| E-3,4,5'-Trihydroxy-3'-Glucopyranosylstilbene | 0.15% |
| E-4,5'-Dihydroxy-3-Methoxy-3'-Glucopyranosylstilbene | 0.81% |
| Eleutheroside B | 0.05% |
| Eriodictyol-7-O-Glucoside | 2.05% |
| Esculin | 0.00% |
| Ethanolamine Phosphate | 0.00% |
| Etidronate | 0.01% |
| Gamma-Glu-Cys | 0.02% |
| Genistein | 0.37% |
| Geranyl Acetone | 0.06% |
| Glucose 6-Phosphate | 0.74% |
| Glycerophosphate(2) | 0.05% |
| Glycolaldehyde Dimer,Mixture Of Stereoisomers | 0.00% |
| Glycyl-L-Proline | 0.01% |
| Glycyrrhizate | 0.38% |
| Guanine | 0.00% |
| Guanosine | 2.13% |
| Guanosine 5'-Diphosphate-D-Mannose | 0.05% |
| Guanosine 5'-Monophosphate | 0.00% |
| Guanosine-3',5'-Cyclic Monophosphate | 0.00% |
| Guanosine-5'-Diphosphate Sodium Salt | 0.31% |
| Guanosine-5'-Triphosphate Sodium Salt | 1.72% |
| Harmaline | 0.00% |
| Heptadecane | 0.00% |
| Hesperetin | 0.00% |
| Hesperetin-7-O-Neohesperidoside | 1.65% |
| Hinokitiol | 0.01% |
| His | 0.01% |
| Histamine | 0.00% |
| Histidinol | 0.00% |
| Hyperoside | 1.24% |
| Hypotaurine | 0.00% |
| Hypoxanthine | 0.03% |
| Indole-3-Acetonitrile | 0.00% |
| Inosine-5'-Diphosphate | 0.84% |
| Inosine-5'-Triphosphate Trisodium Salt | 1.18% |
| Isoguvacine | 0.00% |
| Isopentenyladenine | 0.05% |
| Isorhamnetin-3-O-Rutinoside | 1.15% |
| Isosakuranetin | 0.01% |
| Kaempferol-3-O-(6""-P-Coumaroyl)-Glucoside | 0.41% |
| Kaempferol-3-O-Alpha-L-Arabinoside | 0.05% |
| Kaempferol-3-O-Alpha-L-Rhamnoside | 0.02% |
| L-(-)-Phenylalanine | 0.00% |
| L-(-)-Threonine | 0.00% |
| L-(+)-Lysine | 0.02% |
| L-5-Oxoproline | 0.00% |
| L-Arginine | 0.00% |
| L-Aspartic Acid | 0.00% |
| L-Beta-Homoglutamine-Hcl | 0.00% |
| L-Beta-Homolysine | 0.00% |
| L-Beta-Homomethionine | 0.04% |
| L-Beta-Homothreonine | 0.00% |
| L-Beta-Homotryptophan-Hcl | 0.00% |
| L-Beta-Homotyrosine-Hcl | 0.00% |
| L-Cystine | 0.00% |
| L-Gln | 0.01% |
| L-Homocarnosine | 0.00% |
| L-Iditol | 0.00% |
| L-Leucine | 0.00% |
| L-Methionine | 0.01% |
| L-Methionine Sulfone | 0.00% |
| L-Ornithine | 0.00% |
| L-Proline | 0.00% |
| L-Saccharopine | 0.05% |
| L-Serine | 0.00% |
| Leu-Leu-Tyr | 0.94% |
| Linoleic Acid | 2.36% |
| Luteolin | 0.00% |
| Malate | 0.00% |
| Maleic Acid | 0.00% |
| Malonic Acid | 0.00% |
| Malvidin-3, 5-Di-O-Glucoside Chloride | 0.13% |
| Mecamylamine | 0.03% |
| Melatonin | 0.00% |
| Metformin | 0.00% |
| Methyl Dihydrojasmonate | 0.00% |
| Methyl Jasmonate | 0.00% |
| Methyl Octadecanoate | 0.00% |
| N-6-(Delta-2-Isopentenyl)Adenosinehemihydrate | 1.88% |
| N-Acetyl-D-Mannosamine | 4.11% |
| N-Acetyl-L-Cysteine | 0.22% |
| N-Acetylglutamate | 0.85% |
| N-Acetylglycine | 0.00% |
| N-Acetylputrescine | 0.14% |
| N-Benzoyl(D5) Glycine | 2.30% |
| N-Carbamoyl-L-Aspartic Acid | 0.02% |
| N-Formyl-L-Methionine | 0.00% |
| N-Glycylglycine | 0.00% |
| N-Isovaleroylglycine | 0.00% |
| N-Methylalanine | 0.00% |
| N-Octanoylsphingosine,D-Erythro | 0.12% |
| N-Palmitoyl-D-Erythro-Sphingosine | 0.15% |
| N,N-Dimethylaniline | 0.00% |
| N1-Acetylspermine | 1.11% |
| Nadh | 0.10% |
| Naringenin | 0.00% |
| Nerolidol | 0.00% |
| Nicotinamide | 0.45% |
| Nicotine | 0.09% |
| Nicotinic Acid | 0.00% |
| Norepinephirine | 0.01% |
| Norvaline | 0.00% |
| O-Phenanthroline | 0.00% |
| O-Phosphoserine | 0.00% |
| Ononin | 1.30% |
| Orotic Acid | 0.21% |
| Orth0-Aminobenzoic Acid | 0.00% |
| Oxypurinol | 0.23% |
| P-Hydroxybenzoic Acid | 0.00% |
| Paracetamol | 0.01% |
| Pelargonidin-3-O-Glucoside | 3.33% |
| Petunidin | 0.00% |
| Petunidin-3-O-(6''-O-(4'''-O-E-Coum)-Alpha-Rhamnopyranosyl-Beta-Glucopyranosyl)-5-O-Beta-Glucopyranoside Trifluoroacetate Salt | 1.65% |
| Phenylephrine | 0.58% |
| Phenylhydrazine | 0.00% |
| Phlorizin | 0.03% |
| Phytol | 0.04% |
| Piperacillin Sodium Salt | 2.53% |
| Pterine | 1.91% |
| Pyridoxal 5-Phosphate | 0.23% |
| Pyridoxamine | 0.01% |
| Quercetin | 0.10% |
| Quercetin-3-Arabinoside | 1.36% |
| Quercetin-3-O-Arabinoglucoside | 0.27% |
| Quercetin-3,4'-O-Di-Beta-Glucopyranoside | 0.32% |
| Resveratrol | 0.00% |
| Retinol | 0.00% |
| Ribulose-1,5-Bisphosphate | 3.82% |
| Rosmarinic Acid | 0.41% |
| S-Adenosyl-L-Homocysteine | 0.04% |
| S-Adenosyl-L-Methionine | 0.00% |
| S-Carboxymethylcysteine | 0.06% |
| S-Lactoylglutathione | 1.48% |
| S-Methyl-L-Cysteine | 0.02% |
| Sebacate | 0.14% |
| Sinapyl Alcohol | 0.00% |
| Sissotrin | 0.22% |
| Sodium Deoxycholate | 1.00% |
| Sorbitol 6-Phosphate | 0.84% |
| Spermidine | 0.00% |
| Spermine | 0.67% |
| Syringaldehyde | 0.00% |
| Taurine | 0.01% |
| Tetracosanoic Acid | 0.35% |
| Thiabendazole | 0.87% |
| Thiamine | 0.00% |
| Thymidine | 0.00% |
| Thymidine-3',5'-Cyclic Monophosphate  Sodium Salt | 0.06% |
| Thymidine-5'-Monophosphate | 0.01% |
| Thymine | 0.00% |
| Thymol | 1.32% |
| Trans-4-Hydroxy-L-Proline | 0.00% |
| Trans-Cinnamate | 0.01% |
| Trans-Ortho-Coumaric Acid | 0.01% |
| Trans-Zeatin | 2.25% |
| Trans-Zeatin Riboside | 0.02% |
| Triethanolamine | 0.00% |
| Tropinone | 0.00% |
| Tyr | 0.00% |
| Uracil | 0.00% |
| Uridine | 2.34% |
| Uridine 5'-Monophosphate | 0.00% |
| Uridine-5'-Diphospho-N-Acetylgalactosamine Disodium Salt | 4.21% |
| Uridine-5'-Diphosphogalactose Disodium Salt | 0.19% |
| Urocanic Acid | 0.00% |
| Xanthosine | 0.00% |
| Xanthosine-5'-Monophosphate | 0.00% |
| Zearalenone | 0.00% |
| 3,5-Dibromo-L-Tyrosine | 0.98% |
| 4-Nitrophenyl Phosphate | 0.75% |
| Acacetin-7-O-Rutinoside | 0.04% |
| Adenosine-3',5'-Diphosphate Sodium Salt | 3.18% |
| Benzoic Acid | 0.42% |
| Cyanidin-3-O-(2''-O-Beta-Xylopyranosyl-Beta-Glucopyranoside) | 1.39% |
| Gamma-Tocotrienol | 0.62% |
| Kaempferol-3-O-Robinoside-7-O-Rhamnoside | 2.69% |
| Leupeptin Hemisulfate Salt | 1.78% |
| Peonidin-3,5-O-Di-Beta-Glucopyranoside | 0.73% |
| Rhoifolin | 0.89% |
| Sarsasapogenin | 2.11% |
| Suberic Acid | 3.47% |
| Trans-Zeatin-9-Glucoside | 3.47% |
| Uridine 5'-Diphosphoglucuronic Acid | 0.59% |
| Kynurenic Acid | 2.80% |
| Cyanidin-3, 5-Di-O-Glucoside | 0.15% |
| Indole | 2.42% |
| Anserine | 2.44% |
| Gamma-Terpinene | 0.00% |
| L-Beta-Homoproline | 0.99% |
| Sinapyl Aldehyde | 1.47% |
| 7-Hydroxy-4-Methylcoumarin | 0.07% |

**Appendix 2: The p-values of the Welch's t-test when comparing C vs HC**

| **Metabolites** | **P-Values** |
| --- | --- |
| (-)-Alpha-Copaene | 0.65% |
| (-)-Epicatechin | 0.00% |
| (-)-Riboflavin | 0.17% |
| (+-)-Alpha-Tocopherol Acetateacid Ester | 0.00% |
| (+-)-Jasmonic Acid | 0.00% |
| (+/-)-Cis,Trans-Abscisic Acid | 0.05% |
| 1-Aminocyclopropane-1-Carboxylate | 0.00% |
| 1-Decanoyl-2-Hydroxy-Sn-Glycero-3-Phosphocholine | 2.65% |
| 1-Hexylamine | 0.00% |
| 1-Methylhistamine | 0.04% |
| 1-Myristoyl-2-Hydroxy-Sn-Glycero-3-Phosphate | 0.23% |
| 1-Myristoyl-2-Hydroxy-Sn-Glycero-3-Phosphoethanolamine | 0.14% |
| 1,16-Hexadecanediol | 0.01% |
| 1,2-Dilauroyl-Sn-Glycero-3-Phosphate | 2.30% |
| 1,2-Dipalmitoyl-Sn-Glycero-3-Phospho-Rac-(1-Glycerol) Sodium Salt | 0.36% |
| 1,3-Dimethylurate | 0.00% |
| 1,4-Benzoquinone | 0.00% |
| 12-Oxo-10,15(Z)-Phytodienoic Acid | 2.48% |
| 16-Hydroxyhexadecanoic Acid | 5.49e-08 |
| 1h-Indole-3-Carboxylic Acid | 0.11% |
| 2-(4-Isobutylphenyl)Propionic Acid | 0.11% |
| 2-Mercaptoethanesulfonic Acid | 0.00% |
| 2-Methyllactic Acid | 0.02% |
| 2,3-Diaminopropionate | 0.06% |
| 2,3-Pyridinedicarboxylic Acid | 0.03% |
| 2,5-Dihydroxybenzoic Acid | 0.05% |
| 2'-Deoxyadenosine | 0.00% |
| 2'-Deoxyadenosine 5'-Monophosphate | 0.00% |
| 2'-Deoxycytidine | 0.00% |
| 2'-Deoxyguanosine 5'-Monophosphate | 0.78% |
| 2'-Deoxyinosine | 0.00% |
| 2'-Deoxyinosine 5'-Monophosphate | 0.00% |
| 2'-Deoxyuridine | 0.39% |
| 2'-Deoxyuridine-5'-Monophosphate | 0.00% |
| 3 5 7-Trihydroxy-4'-Methoxyflavone | 0.00% |
| 3-(2-Aminoethyl)Indole | 2.43% |
| 3-(4-Hydroxy-3-Methoxyphenyl)Prop-2-Enoicacid | 0.00% |
| 3-(4-Hydroxy-3,5-Dimethoxyphenyl)-2-Propenoic Acid | 0.01% |
| 3-(4-Hydroxyphenyl)Propionic Acid | 0.01% |
| 3-(Methylsulfinyl)Propylglucosinolate | 0.03% |
| 3-Chloro-L-Tyrosine | 0.22% |
| 3-Formylindole | 0.01% |
| 3-Hydroxy-3-Methylglutaric Acid | 0.06% |
| 3-Hydroxyanthranilic Acid | 0.00% |
| 3-Hydroxyisovaleric Acid | 0.00% |
| 3-Methylxanthine | 0.02% |
| 3-Phospho-D-Glycerate | 0.06% |
| 3,4-Dihydroxy-L-Phenylalanine | 0.00% |
| 3,4-Dihydroxymandelate | 0.73% |
| 3,4-Dimethoxycinnamic Acid | 0.25% |
| 3' 4' 5 7-Tetrahydroxyflavanone | 0.00% |
| 3'-Dephosphocoenzyme A | 0.23% |
| 3'-Methoxy-4',5,7-Trihydroxyflavonol | 0.00% |
| 4-(2 Aminoethyl)-Phenol | 0.00% |
| 4-Aminophenol | 0.00% |
| 4-Hydroxy-3-Methoxycinnamaldehyde | 0.00% |
| 4-Hydroxyphenylpyruvic Acid | 0.00% |
| 4-Methyl-5-Thiazoleethanol | 0.00% |
| 4-Methylsulfinylbutyl Glucosinolate | 3.18% |
| 4-Nonanolide | 0.03% |
| 4-Pyridoxate | 0.00% |
| 5-Aminoimidazole-4-Carboxamide-1-Ribofuranosyl 5'-Monophosphate | 0.25% |
| 5-Butylpyridine-2-Carboxylic Acid | 0.00% |
| 5-Hydroxyindoleacetic Acid | 4.65% |
| 6-Hydroxynicotinic Acid | 0.00% |
| 7-Acetoxy-4-Methylcoumarin | 0.00% |
| A-Lipoamide | 3.73% |
| Acacetin | 0.78% |
| Adenine | 0.05% |
| Adenosine | 0.36% |
| Adenosine 3':5'-Cyclicmonophosphate | 0.00% |
| Adenosine 5'-Diphospho-Glucose | 0.41% |
| Agmatine | 0.02% |
| All-Trans-Retinoic Acid | 0.00% |
| Allantoin | 0.00% |
| Alpha-D-Glucose-1,6-Diphosphate | 0.02% |
| Alpha-L-(-)-Fucose 1-Phosphate Bis(Cyclohexylammonium) Salt | 0.03% |
| Alpha-Methyl-Dl-Histidine | 0.01% |
| Alpha-Tocotrienol | 0.06% |
| Amantadine | 0.00% |
| Baclofen | 0.07% |
| Baicalein-7-O-Glucuronide | 2.55% |
| Benzaldehyde | 0.00% |
| Benzamidine | 0.07% |
| Beta-Nicotinamide Mononucleotide | 0.00% |
| Cadaverine | 0.00% |
| Caffeine | 0.07% |
| Calciferol | 1.17% |
| Camphene | 0.00% |
| Canavanine | 0.00% |
| Canthaxanthin | 0.14% |
| Capsaicin | 0.01% |
| Carnosine | 0.00% |
| Cerulenin | 0.00% |
| Chalcone | 0.00% |
| Chlorogenic Acid | 4.60% |
| Cholecalciferol | 0.25% |
| Choline | 0.00% |
| Cis-Aconitate | 0.09% |
| Cis,Cis-Muconic Acid | 0.00% |
| Citraconic Acid | 0.00% |
| Citramalate | 0.00% |
| Citrulline | 0.00% |
| Creatinine | 0.00% |
| Crotonoyl Coenzyme A Lithium Salt | 0.54% |
| Cyanidin-3-O-(6''-O-(E-P-Coum)-2''-O-(Beta-Xylopyranosyl)-Beta-Glucopyranoside)-5-O-Beta-Glucopyranoside | 0.06% |
| Cyanidin-3-O-Galactoside | 0.21% |
| Cysteine S-Sulfate | 0.07% |
| Cytidine | 0.04% |
| Cytidine 5'-Diphosphocholine | 0.41% |
| Cytidine-3',5'-Cyclicmonophosphate | 0.01% |
| Cytidine-5'-Monophosphate | 0.01% |
| Cytosine | 0.00% |
| D-(+)-Maltose | 0.02% |
| D-Carnitine | 0.00% |
| D-Cysteine | 0.04% |
| D-Ribose 5-Phosphate | 0.00% |
| D,L-Sulforaphane | 0.15% |
| Daidzein | 0.00% |
| Daphnetin | 0.14% |
| Delta-Tocopherol | 0.49% |
| Delta-Tocotrienol | 0.25% |
| Diethanolamine | 0.02% |
| Dihydrocapsaicin | 0.00% |
| Dihydrosphingosine | 0.00% |
| Dihydrouracil | 0.00% |
| Diosmin | 1.88% |
| Dl-5-Hydroxylysine | 0.95% |
| Dl-Alpha,Epsilon-Diaminopimelic Acid | 0.00% |
| Dl-Cystathionine | 0.00% |
| Dl-Dihydrozeatin | 0.01% |
| Dl-Threo-Beta-Methylaspartic Acid | 0.00% |
| Dudp | 1.27% |
| E-3,4,5'-Trihydroxy-3'-Glucopyranosylstilbene | 0.26% |
| Eleutheroside B | 0.03% |
| Eriodictyol-7-O-Glucoside | 0.33% |
| Esculin | 0.00% |
| Ethanolamine Phosphate | 0.00% |
| Etidronate | 0.01% |
| Gamma-Glu-Cys | 0.02% |
| Genistein | 0.46% |
| Geranyl Acetone | 0.14% |
| Glucose 6-Phosphate | 0.76% |
| Glycerophosphate(2) | 0.04% |
| Glycolaldehyde Dimer,Mixture Of Stereoisomers | 0.01% |
| Glycyl-L-Proline | 0.01% |
| Glycyrrhizate | 0.72% |
| Guanine | 0.00% |
| Guanosine 5'-Diphosphate-D-Mannose | 0.06% |
| Guanosine 5'-Monophosphate | 3.35% |
| Guanosine-3',5'-Cyclic Monophosphate | 0.00% |
| Guanosine-5'-Diphosphate Sodium Salt | 0.00% |
| Harmaline | 0.00% |
| Heptadecane | 0.00% |
| Hesperetin | 0.00% |
| Hesperetin-7-O-Neohesperidoside | 0.79% |
| Hinokitiol | 0.01% |
| His | 0.04% |
| Histamine | 0.00% |
| Histidinol | 0.00% |
| Hypotaurine | 0.00% |
| Hypoxanthine | 0.01% |
| Indole-3-Acetonitrile | 0.00% |
| Inosine | 2.92% |
| Inosine-5'-Diphosphate | 0.41% |
| Inosine-5'-Triphosphate Trisodium Salt | 2.35% |
| Isoguvacine | 0.00% |
| Isopentenyladenine | 0.06% |
| Isorhamnetin-3-O-Rutinoside | 4.59% |
| Isosakuranetin | 0.01% |
| Kaempferol-3-O-Alpha-L-Rhamnoside | 0.03% |
| L-(-)-Phenylalanine | 0.00% |
| L-(-)-Threonine | 0.04% |
| L-(+)-Lysine | 0.07% |
| L-5-Oxoproline | 0.00% |
| L-Arginine | 0.00% |
| L-Aspartic Acid | 0.00% |
| L-Beta-Homoglutamine-Hcl | 0.00% |
| L-Beta-Homolysine | 0.00% |
| L-Beta-Homomethionine | 0.06% |
| L-Beta-Homothreonine | 0.00% |
| L-Beta-Homotryptophan-Hcl | 4.51% |
| L-Beta-Homotyrosine-Hcl | 0.00% |
| L-Cystine | 0.00% |
| L-Gln | 0.01% |
| L-Homocarnosine | 0.00% |
| L-Iditol | 0.00% |
| L-Leucine | 0.00% |
| L-Methionine | 0.01% |
| L-Methionine Sulfone | 0.00% |
| L-Ornithine | 0.00% |
| L-Proline | 0.00% |
| L-Saccharopine | 0.05% |
| L-Serine | 0.00% |
| Leu-Leu-Tyr | 1.48% |
| Linoleic Acid | 0.78% |
| Luteolin | 0.00% |
| Luteolin-7-O-Glucoside | 4.68% |
| Malate | 0.00% |
| Maleic Acid | 0.00% |
| Malonic Acid | 0.01% |
| Malvidin-3, 5-Di-O-Glucoside Chloride | 0.12% |
| Mecamylamine | 0.02% |
| Melatonin | 0.23% |
| Metformin | 0.00% |
| Methyl Dihydrojasmonate | 0.00% |
| Methyl Jasmonate | 0.00% |
| Methyl Octadecanoate | 0.00% |
| N-Acetyl-L-Cysteine | 0.33% |
| N-Acetylglutamate | 0.48% |
| N-Acetylglycine | 0.00% |
| N-Acetylputrescine | 0.33% |
| N-Benzoyl(D5) Glycine | 2.18% |
| N-Carbamoyl-L-Aspartic Acid | 0.09% |
| N-Formyl-L-Methionine | 0.00% |
| N-Glycylglycine | 0.00% |
| N-Isovaleroylglycine | 0.00% |
| N-Methylalanine | 0.00% |
| N-Octanoylsphingosine,D-Erythro | 4.78% |
| N-Palmitoyl-D-Erythro-Sphingosine | 0.00% |
| N,N-Dimethylaniline | 0.05% |
| N1-Acetylspermine | 1.21% |
| Nadh | 0.21% |
| Naringenin | 0.00% |
| Nerolidol | 0.00% |
| Nicotinamide | 1.19% |
| Nicotine | 0.03% |
| Nicotinic Acid | 0.00% |
| Norepinephirine | 0.01% |
| Norvaline | 0.00% |
| O-Phenanthroline | 0.00% |
| O-Phosphoserine | 0.00% |
| Ononin | 1.63% |
| Orotic Acid | 0.28% |
| Orth0-Aminobenzoic Acid | 0.00% |
| Oxypurinol | 0.19% |
| P-Hydroxybenzoic Acid | 0.00% |
| Paracetamol | 0.01% |
| Petunidin | 0.00% |
| Petunidin-3-O-(6''-O-(4'''-O-E-Coum)-Alpha-Rhamnopyranosyl-Beta-Glucopyranosyl)-5-O-Beta-Glucopyranoside Trifluoroacetate Salt | 1.30% |
| Phenylephrine | 0.37% |
| Phenylhydrazine | 0.00% |
| Phlorizin | 0.02% |
| Phytol | 0.01% |
| Piperacillin Sodium Salt | 0.80% |
| Pterine | 3.68% |
| Pyridoxal 5-Phosphate | 0.23% |
| Quercetin | 0.04% |
| Quercetin-3-O-Arabinoglucoside | 0.32% |
| Quercetin-3,4'-O-Di-Beta-Glucopyranoside | 0.37% |
| Resveratrol | 0.00% |
| Retinol | 0.00% |
| Ribulose-1,5-Bisphosphate | 3.68% |
| S-Adenosyl-L-Methionine | 0.00% |
| S-Carboxymethylcysteine | 0.04% |
| S-Lactoylglutathione | 1.83% |
| S-Methyl-L-Cysteine | 0.02% |
| Scopoletin | 0.00% |
| Sebacate | 0.12% |
| Sinapyl Alcohol | 0.00% |
| Sissotrin | 0.12% |
| Sodium Deoxycholate | 0.49% |
| Sorbitol 6-Phosphate | 0.75% |
| Spermidine | 0.01% |
| Spermine | 0.68% |
| Syringaldehyde | 0.00% |
| Taurine | 0.01% |
| Tetracosanoic Acid | 0.18% |
| Thiabendazole | 0.89% |
| Thiamine | 0.00% |
| Thymidine | 0.00% |
| Thymidine-3',5'-Cyclic Monophosphate  Sodium Salt | 0.01% |
| Thymidine-5'-Monophosphate | 0.12% |
| Thymine | 0.00% |
| Thymol | 0.60% |
| Trans-4-Hydroxy-L-Proline | 0.00% |
| Trans-Cinnamate | 3.56% |
| Trans-Ortho-Coumaric Acid | 0.01% |
| Trans-Zeatin Riboside | 0.15% |
| Triethanolamine | 0.01% |
| Tropinone | 0.00% |
| Tyr | 0.00% |
| Uracil | 0.00% |
| Uridine | 2.35% |
| Uridine 5'-Monophosphate | 0.00% |
| Uridine-5'-Diphosphogalactose Disodium Salt | 1.84% |
| Urocanic Acid | 0.00% |
| Xanthosine | 0.00% |
| Xanthosine-5'-Monophosphate | 0.06% |
| Zearalenone | 0.00% |
| 3,5-Dibromo-L-Tyrosine | 1.13% |
| 4-Nitrophenyl Phosphate | 1.75% |
| Acacetin-7-O-Rutinoside | 0.05% |
| Benzoic Acid | 2.33% |
| Cyanidin-3-O-(2''-O-Beta-Xylopyranosyl-Beta-Glucopyranoside) | 0.68% |
| Cytidine-5'-Triphosphate Disodium Salt | 2.32% |
| Gamma-Tocotrienol | 0.31% |
| Kaempferol-3-Glucuronide | 1.32% |
| Leupeptin Hemisulfate Salt | 0.15% |
| Peonidin-3,5-O-Di-Beta-Glucopyranoside | 1.02% |
| Rhoifolin | 1.39% |
| Sarsasapogenin | 0.03% |
| Solasodine | 3.42% |
| Suberic Acid | 4.45% |
| Uridine 5'-Diphosphoglucuronic Acid | 0.67% |
| N-Propionyl Coenzyme A Lithium Salt | 4.70% |
| Isosakuranetin-7-O-Neohesperidoside | 1.49% |
| Kynurenic Acid | 1.53% |
| Cyanidin-3, 5-Di-O-Glucoside | 0.05% |
| Gamma-Terpinene | 0.01% |
| Sinapyl Aldehyde | 3.80% |

**Appendix 3: The p-values of the Welch's t-test when comparing P vs HC**

| **Metabolites** | **P-Values** |
| --- | --- |
| (+-)-Jasmonic Acid | 4.45% |
| 1,16-Hexadecanediol | 3.65% |
| 16-Hydroxyhexadecanoic Acid | 2.63% |
| 2-Methyllactic Acid | 0.42% |
| 2'-Deoxyinosine 5'-Monophosphate | 2.22% |
| 4-Methylsulfinylbutyl Glucosinolate | 3.70% |
| 4-Nonanolide | 4.16% |
| Beta-Nicotinamide Mononucleotide | 2.85% |
| Calciferol | 3.63% |
| Cinnamyl Alcohol | 2.60% |
| Creatinine | 3.79% |
| Cytidine | 1.60% |
| D-Glucosyl-Beta1-1'-D-Erythoro-Sphingosine | 1.75% |
| Daphnetin | 0.80% |
| Dl-Cystathionine | 0.25% |
| Gamma-Glu-Cys | 2.69% |
| His | 4.65% |
| Histamine | 2.55% |
| N-Palmitoyl-D-Erythro-Sphingosine | 3.03% |
| Nicotine | 2.80% |
| Phytol | 4.53% |
| Quercetin | 0.27% |
| Sorbitol 6-Phosphate | 0.52% |
| Leupeptin Hemisulfate Salt | 3.00% |
| Sarsasapogenin | 0.62% |
| L-Beta-Homoproline | 4.04% |

**Appendix 4: Significantly altered metabolites between C and P after Bonferroni Correction**

| **Metabolites** | **P-Values** |
| --- | --- |
| Gamma-Terpinene | 0.00% |
| Spermidine | 0.00% |
| Metformin | 0.00% |
| 1-Hexylamine | 0.00% |
| Resveratrol | 0.00% |
| Methyl Octadecanoate | 0.00% |
| Allantoin | 0.00% |
| Dl-Alpha,Epsilon-Diaminopimelic Acid | 0.00% |
| N-Glycylglycine | 0.00% |
| Thymine | 0.00% |
| 2'-Deoxyadenosine | 0.00% |
| Cerulenin | 0.00% |
| Alpha-Methyl-Dl-Histidine | 0.00% |
| 2,3-Diaminopropionate | 0.00% |
| Hesperetin | 0.00% |
| L-Leucine | 0.00% |
| Dihydrosphingosine | 0.00% |
| S-Adenosyl-L-Methionine | 0.00% |
| Daidzein | 0.00% |
| Adenosine 3':5'-Cyclicmonophosphate | 0.00% |
| Norvaline | 0.00% |
| Uracil | 0.00% |
| Adenine | 0.00% |
| O-Phenanthroline | 0.00% |
| Dl-Threo-Beta-Methylaspartic Acid | 0.00% |
| Urocanic Acid | 0.00% |
| L-5-Oxoproline | 0.00% |
| Cytosine | 0.00% |
| Dihydrouracil | 0.00% |
| 16-Hydroxyhexadecanoic Acid | 0.00% |
| Histamine | 0.00% |
| Xanthosine | 0.00% |
| Cadaverine | 0.00% |
| Nerolidol | 0.00% |
| 1,4-Benzoquinone | 0.00% |
| N-Acetylglycine | 0.00% |
| L-Homocarnosine | 0.00% |
| Creatinine | 0.00% |
| P-Hydroxybenzoic Acid | 0.00% |
| L-Beta-Homotyrosine-Hcl | 0.00% |
| 4-(2 Aminoethyl)-Phenol | 0.00% |
| Hypotaurine | 0.00% |
| Isoguvacine | 0.00% |
| Naringenin | 0.00% |
| Heptadecane | 0.00% |
| Retinol | 0.00% |
| L-Aspartic Acid | 0.00% |
| Chalcone | 0.00% |
| Melatonin | 0.00% |
| 1,3-Dimethylurate | 0.00% |
| 1-Aminocyclopropane-1-Carboxylate | 0.00% |
| N-Isovaleroylglycine | 0.00% |
| D-Ribose 5-Phosphate | 0.00% |
| L-(-)-Threonine | 0.00% |
| Camphene | 0.00% |
| 2'-Deoxyuridine-5'-Monophosphate | 0.00% |
| Ethanolamine Phosphate | 0.00% |
| Luteolin | 0.00% |
| Maleic Acid | 0.00% |
| Sinapyl Alcohol | 0.00% |
| 4-Aminophenol | 0.00% |
| 3,4-Dihydroxy-L-Phenylalanine | 0.00% |
| 3 5 7-Trihydroxy-4'-Methoxyflavone | 0.00% |
| L-Beta-Homothreonine | 0.00% |
| 6-Hydroxynicotinic Acid | 0.00% |
| Carnosine | 0.00% |
| O-Phosphoserine | 0.00% |
| Amantadine | 0.00% |
| L-Beta-Homolysine | 0.00% |
| (+-)-Jasmonic Acid | 0.00% |
| L-(-)-Phenylalanine | 0.00% |
| Benzaldehyde | 0.00% |
| Phenylhydrazine | 0.00% |
| Canavanine | 0.00% |
| Uridine 5'-Monophosphate | 0.00% |
| L-Ornithine | 0.00% |
| Xanthosine-5'-Monophosphate | 0.00% |
| D-Carnitine | 0.00% |
| Esculin | 0.00% |
| Orth0-Aminobenzoic Acid | 0.00% |
| Dihydrocapsaicin | 0.00% |
| 2'-Deoxyinosine 5'-Monophosphate | 0.00% |
| Tropinone | 0.00% |
| Malonic Acid | 0.00% |
| 2'-Deoxyadenosine 5'-Monophosphate | 0.00% |
| Malate | 0.00% |
| 3-Hydroxyisovaleric Acid | 0.00% |
| Choline | 0.00% |
| N,N-Dimethylaniline | 0.00% |
| Nicotinic Acid | 0.00% |
| 3' 4' 5 7-Tetrahydroxyflavanone | 0.00% |
| N-Methylalanine | 0.00% |
| 2'-Deoxyinosine | 0.00% |
| Syringaldehyde | 0.00% |
| L-Methionine Sulfone | 0.00% |
| 3-(4-Hydroxy-3-Methoxyphenyl)Prop-2-Enoicacid | 0.00% |
| Petunidin | 0.00% |
| 3-Hydroxyanthranilic Acid | 0.00% |
| Thiamine | 0.00% |
| L-Serine | 0.00% |
| 4-Methyl-5-Thiazoleethanol | 0.00% |
| N-Formyl-L-Methionine | 0.00% |
| Guanosine-3',5'-Cyclic Monophosphate | 0.00% |
| Tyr | 0.00% |
| (-)-Epicatechin | 0.00% |
| L-Beta-Homotryptophan-Hcl | 0.00% |
| Trans-4-Hydroxy-L-Proline | 0.00% |
| Methyl Dihydrojasmonate | 0.00% |
| Guanosine 5'-Monophosphate | 0.00% |
| Histidinol | 0.00% |
| Indole-3-Acetonitrile | 0.00% |
| Methyl Jasmonate | 0.00% |
| Zearalenone | 0.00% |
| Cis,Cis-Muconic Acid | 0.00% |
| 3-Formylindole | 0.00% |
| Diethanolamine | 0.00% |
| 4-Hydroxy-3-Methoxycinnamaldehyde | 0.00% |
| L-Beta-Homoglutamine-Hcl | 0.00% |
| Citraconic Acid | 0.00% |
| L-Proline | 0.00% |
| 2-Mercaptoethanesulfonic Acid | 0.00% |
| L-Iditol | 0.00% |
| L-Cystine | 0.00% |
| L-Arginine | 0.00% |
| 3'-Methoxy-4',5,7-Trihydroxyflavonol | 0.00% |
| Citrulline | 0.00% |
| Harmaline | 0.00% |
| Citramalate | 0.00% |
| 4-Pyridoxate | 0.00% |
| 2'-Deoxycytidine | 0.00% |
| All-Trans-Retinoic Acid | 0.00% |
| Agmatine | 0.00% |
| 4-Hydroxyphenylpyruvic Acid | 0.00% |
| Guanine | 0.00% |
| Thymidine | 0.00% |
| Glycolaldehyde Dimer,Mixture Of Stereoisomers | 0.00% |
| Triethanolamine | 0.00% |
| (-)-Riboflavin | 0.00% |
| Trans-Cinnamate | 0.01% |
| Cytidine-3',5'-Cyclicmonophosphate | 0.01% |
| Norepinephirine | 0.01% |
| (+-)-Alpha-Tocopherol Acetateacid Ester | 0.01% |
| 4-Nonanolide | 0.01% |
| Dl-Cystathionine | 0.01% |
| L-Gln | 0.01% |
| Dl-Dihydrozeatin | 0.01% |
| Isosakuranetin | 0.01% |
| Paracetamol | 0.01% |
| 3-(4-Hydroxy-3,5-Dimethoxyphenyl)-2-Propenoic Acid | 0.01% |
| His | 0.01% |
| Hinokitiol | 0.01% |
| Taurine | 0.01% |
| Trans-Ortho-Coumaric Acid | 0.01% |
| Thymidine-5'-Monophosphate | 0.01% |
| 1-Methylhistamine | 0.01% |

**Appendix 5: Significantly altered metabolites between C and P after Holm-Bonferroni Correction**

| **Metabolites** | **P-Values** |
| --- | --- |
| Gamma-Terpinene | 0.00% |
| Spermidine | 0.00% |
| Metformin | 0.00% |
| 1-Hexylamine | 0.00% |
| Resveratrol | 0.00% |
| Methyl Octadecanoate | 0.00% |
| Allantoin | 0.00% |
| Dl-Alpha,Epsilon-Diaminopimelic Acid | 0.00% |
| N-Glycylglycine | 0.00% |
| Thymine | 0.00% |
| 2'-Deoxyadenosine | 0.00% |
| Cerulenin | 0.00% |
| Alpha-Methyl-Dl-Histidine | 0.00% |
| 2,3-Diaminopropionate | 0.00% |
| Hesperetin | 0.00% |
| L-Leucine | 0.00% |
| Dihydrosphingosine | 0.00% |
| S-Adenosyl-L-Methionine | 0.00% |
| Daidzein | 0.00% |
| Adenosine 3':5'-Cyclicmonophosphate | 0.00% |
| Norvaline | 0.00% |
| Uracil | 0.00% |
| Adenine | 0.00% |
| O-Phenanthroline | 0.00% |
| Dl-Threo-Beta-Methylaspartic Acid | 0.00% |
| Urocanic Acid | 0.00% |
| L-5-Oxoproline | 0.00% |
| Cytosine | 0.00% |
| Dihydrouracil | 0.00% |
| 16-Hydroxyhexadecanoic Acid | 0.00% |
| Histamine | 0.00% |
| Xanthosine | 0.00% |
| Cadaverine | 0.00% |
| Nerolidol | 0.00% |
| 1,4-Benzoquinone | 0.00% |
| N-Acetylglycine | 0.00% |
| L-Homocarnosine | 0.00% |
| Creatinine | 0.00% |
| P-Hydroxybenzoic Acid | 0.00% |
| L-Beta-Homotyrosine-Hcl | 0.00% |
| 4-(2 Aminoethyl)-Phenol | 0.00% |
| Hypotaurine | 0.00% |
| Isoguvacine | 0.00% |
| Naringenin | 0.00% |
| Heptadecane | 0.00% |
| Retinol | 0.00% |
| L-Aspartic Acid | 0.00% |
| Chalcone | 0.00% |
| Melatonin | 0.00% |
| 1,3-Dimethylurate | 0.00% |
| 1-Aminocyclopropane-1-Carboxylate | 0.00% |
| N-Isovaleroylglycine | 0.00% |
| D-Ribose 5-Phosphate | 0.00% |
| L-(-)-Threonine | 0.00% |
| Camphene | 0.00% |
| 2'-Deoxyuridine-5'-Monophosphate | 0.00% |
| Ethanolamine Phosphate | 0.00% |
| Luteolin | 0.00% |
| Maleic Acid | 0.00% |
| Sinapyl Alcohol | 0.00% |
| 4-Aminophenol | 0.00% |
| 3,4-Dihydroxy-L-Phenylalanine | 0.00% |
| 3 5 7-Trihydroxy-4'-Methoxyflavone | 0.00% |
| L-Beta-Homothreonine | 0.00% |
| 6-Hydroxynicotinic Acid | 0.00% |
| Carnosine | 0.00% |
| O-Phosphoserine | 0.00% |
| Amantadine | 0.00% |
| L-Beta-Homolysine | 0.00% |
| (+-)-Jasmonic Acid | 0.00% |
| L-(-)-Phenylalanine | 0.00% |
| Benzaldehyde | 0.00% |
| Phenylhydrazine | 0.00% |
| Canavanine | 0.00% |
| Uridine 5'-Monophosphate | 0.00% |
| L-Ornithine | 0.00% |
| Xanthosine-5'-Monophosphate | 0.00% |
| D-Carnitine | 0.00% |
| Esculin | 0.00% |
| Orth0-Aminobenzoic Acid | 0.00% |
| Dihydrocapsaicin | 0.00% |
| 2'-Deoxyinosine 5'-Monophosphate | 0.00% |
| Tropinone | 0.00% |
| Malonic Acid | 0.00% |
| 2'-Deoxyadenosine 5'-Monophosphate | 0.00% |
| Malate | 0.00% |
| 3-Hydroxyisovaleric Acid | 0.00% |
| Choline | 0.00% |
| N,N-Dimethylaniline | 0.00% |
| Nicotinic Acid | 0.00% |
| 3' 4' 5 7-Tetrahydroxyflavanone | 0.00% |
| N-Methylalanine | 0.00% |
| 2'-Deoxyinosine | 0.00% |
| Syringaldehyde | 0.00% |
| L-Methionine Sulfone | 0.00% |
| 3-(4-Hydroxy-3-Methoxyphenyl)Prop-2-Enoicacid | 0.00% |
| Petunidin | 0.00% |
| 3-Hydroxyanthranilic Acid | 0.00% |
| Thiamine | 0.00% |
| L-Serine | 0.00% |
| 4-Methyl-5-Thiazoleethanol | 0.00% |
| N-Formyl-L-Methionine | 0.00% |
| Guanosine-3',5'-Cyclic Monophosphate | 0.00% |
| Tyr | 0.00% |
| (-)-Epicatechin | 0.00% |
| L-Beta-Homotryptophan-Hcl | 0.00% |
| Trans-4-Hydroxy-L-Proline | 0.00% |
| Methyl Dihydrojasmonate | 0.00% |
| Guanosine 5'-Monophosphate | 0.00% |
| Histidinol | 0.00% |
| Indole-3-Acetonitrile | 0.00% |
| Methyl Jasmonate | 0.00% |
| Zearalenone | 0.00% |
| Cis,Cis-Muconic Acid | 0.00% |
| 3-Formylindole | 0.00% |
| Diethanolamine | 0.00% |
| 4-Hydroxy-3-Methoxycinnamaldehyde | 0.00% |
| L-Beta-Homoglutamine-Hcl | 0.00% |
| Citraconic Acid | 0.00% |
| L-Proline | 0.00% |
| 2-Mercaptoethanesulfonic Acid | 0.00% |
| L-Iditol | 0.00% |
| L-Cystine | 0.00% |
| L-Arginine | 0.00% |
| 3'-Methoxy-4',5,7-Trihydroxyflavonol | 0.00% |
| Citrulline | 0.00% |
| Harmaline | 0.00% |
| Citramalate | 0.00% |
| 4-Pyridoxate | 0.00% |
| 2'-Deoxycytidine | 0.00% |
| All-Trans-Retinoic Acid | 0.00% |
| Agmatine | 0.00% |
| 4-Hydroxyphenylpyruvic Acid | 0.00% |
| Guanine | 0.00% |
| Thymidine | 0.00% |
| Glycolaldehyde Dimer,Mixture Of Stereoisomers | 0.00% |
| Triethanolamine | 0.00% |
| (-)-Riboflavin | 0.00% |
| Trans-Cinnamate | 0.01% |
| Cytidine-3',5'-Cyclicmonophosphate | 0.01% |
| Norepinephirine | 0.01% |
| (+-)-Alpha-Tocopherol Acetateacid Ester | 0.01% |
| 4-Nonanolide | 0.01% |
| Dl-Cystathionine | 0.01% |
| L-Gln | 0.01% |
| Dl-Dihydrozeatin | 0.01% |
| Isosakuranetin | 0.01% |
| Paracetamol | 0.01% |
| 3-(4-Hydroxy-3,5-Dimethoxyphenyl)-2-Propenoic Acid | 0.01% |
| His | 0.01% |
| Hinokitiol | 0.01% |
| Taurine | 0.01% |
| Trans-Ortho-Coumaric Acid | 0.01% |
| Thymidine-5'-Monophosphate | 0.01% |
| 1-Methylhistamine | 0.01% |
| Etidronate | 0.01% |
| L-Methionine | 0.01% |
| 1,16-Hexadecanediol | 0.01% |
| Glycyl-L-Proline | 0.01% |
| 5-Butylpyridine-2-Carboxylic Acid | 0.01% |
| Pyridoxamine | 0.01% |
| Alpha-D-Glucose-1,6-Diphosphate | 0.01% |
| Cytidine-5'-Monophosphate | 0.02% |
| Capsaicin | 0.02% |
| S-Methyl-L-Cysteine | 0.02% |
| Gamma-Glu-Cys | 0.02% |
| 3-(4-Hydroxyphenyl)Propionic Acid | 0.02% |
| Cholecalciferol | 0.02% |

**Appendix 6: Significantly altered metabolites between C and P after Benjamini-Hochberg Correction**

| **Metabolites** | **P-Values** |
| --- | --- |
| Gamma-Terpinene | 0.00% |
| Spermidine | 0.00% |
| Metformin | 0.00% |
| 1-Hexylamine | 0.00% |
| Resveratrol | 0.00% |
| Methyl Octadecanoate | 0.00% |
| Allantoin | 0.00% |
| Dl-Alpha,Epsilon-Diaminopimelic Acid | 0.00% |
| N-Glycylglycine | 0.00% |
| Thymine | 0.00% |
| 2'-Deoxyadenosine | 0.00% |
| Cerulenin | 0.00% |
| Alpha-Methyl-Dl-Histidine | 0.00% |
| 2,3-Diaminopropionate | 0.00% |
| Hesperetin | 0.00% |
| L-Leucine | 0.00% |
| Dihydrosphingosine | 0.00% |
| S-Adenosyl-L-Methionine | 0.00% |
| Daidzein | 0.00% |
| Adenosine 3':5'-Cyclicmonophosphate | 0.00% |
| Norvaline | 0.00% |
| Uracil | 0.00% |
| Adenine | 0.00% |
| O-Phenanthroline | 0.00% |
| Dl-Threo-Beta-Methylaspartic Acid | 0.00% |
| Urocanic Acid | 0.00% |
| L-5-Oxoproline | 0.00% |
| Cytosine | 0.00% |
| Dihydrouracil | 0.00% |
| 16-Hydroxyhexadecanoic Acid | 0.00% |
| Histamine | 0.00% |
| Xanthosine | 0.00% |
| Cadaverine | 0.00% |
| Nerolidol | 0.00% |
| 1,4-Benzoquinone | 0.00% |
| N-Acetylglycine | 0.00% |
| L-Homocarnosine | 0.00% |
| Creatinine | 0.00% |
| P-Hydroxybenzoic Acid | 0.00% |
| L-Beta-Homotyrosine-Hcl | 0.00% |
| 4-(2 Aminoethyl)-Phenol | 0.00% |
| Hypotaurine | 0.00% |
| Isoguvacine | 0.00% |
| Naringenin | 0.00% |
| Heptadecane | 0.00% |
| Retinol | 0.00% |
| L-Aspartic Acid | 0.00% |
| Chalcone | 0.00% |
| Melatonin | 0.00% |
| 1,3-Dimethylurate | 0.00% |
| 1-Aminocyclopropane-1-Carboxylate | 0.00% |
| N-Isovaleroylglycine | 0.00% |
| D-Ribose 5-Phosphate | 0.00% |
| L-(-)-Threonine | 0.00% |
| Camphene | 0.00% |
| 2'-Deoxyuridine-5'-Monophosphate | 0.00% |
| Ethanolamine Phosphate | 0.00% |
| Luteolin | 0.00% |
| Maleic Acid | 0.00% |
| Sinapyl Alcohol | 0.00% |
| 4-Aminophenol | 0.00% |
| 3,4-Dihydroxy-L-Phenylalanine | 0.00% |
| 3 5 7-Trihydroxy-4'-Methoxyflavone | 0.00% |
| L-Beta-Homothreonine | 0.00% |
| 6-Hydroxynicotinic Acid | 0.00% |
| Carnosine | 0.00% |
| O-Phosphoserine | 0.00% |
| Amantadine | 0.00% |
| L-Beta-Homolysine | 0.00% |
| (+-)-Jasmonic Acid | 0.00% |
| L-(-)-Phenylalanine | 0.00% |
| Benzaldehyde | 0.00% |
| Phenylhydrazine | 0.00% |
| Canavanine | 0.00% |
| Uridine 5'-Monophosphate | 0.00% |
| L-Ornithine | 0.00% |
| Xanthosine-5'-Monophosphate | 0.00% |
| D-Carnitine | 0.00% |
| Esculin | 0.00% |
| Orth0-Aminobenzoic Acid | 0.00% |
| Dihydrocapsaicin | 0.00% |
| 2'-Deoxyinosine 5'-Monophosphate | 0.00% |
| Tropinone | 0.00% |
| Malonic Acid | 0.00% |
| 2'-Deoxyadenosine 5'-Monophosphate | 0.00% |
| Malate | 0.00% |
| 3-Hydroxyisovaleric Acid | 0.00% |
| Choline | 0.00% |
| N,N-Dimethylaniline | 0.00% |
| Nicotinic Acid | 0.00% |
| 3' 4' 5 7-Tetrahydroxyflavanone | 0.00% |
| N-Methylalanine | 0.00% |
| 2'-Deoxyinosine | 0.00% |
| Syringaldehyde | 0.00% |
| L-Methionine Sulfone | 0.00% |
| 3-(4-Hydroxy-3-Methoxyphenyl)Prop-2-Enoicacid | 0.00% |
| Petunidin | 0.00% |
| 3-Hydroxyanthranilic Acid | 0.00% |
| Thiamine | 0.00% |
| L-Serine | 0.00% |
| 4-Methyl-5-Thiazoleethanol | 0.00% |
| N-Formyl-L-Methionine | 0.00% |
| Guanosine-3',5'-Cyclic Monophosphate | 0.00% |
| Tyr | 0.00% |
| (-)-Epicatechin | 0.00% |
| L-Beta-Homotryptophan-Hcl | 0.00% |
| Trans-4-Hydroxy-L-Proline | 0.00% |
| Methyl Dihydrojasmonate | 0.00% |
| Guanosine 5'-Monophosphate | 0.00% |
| Histidinol | 0.00% |
| Indole-3-Acetonitrile | 0.00% |
| Methyl Jasmonate | 0.00% |
| Zearalenone | 0.00% |
| Cis,Cis-Muconic Acid | 0.00% |
| 3-Formylindole | 0.00% |
| Diethanolamine | 0.00% |
| 4-Hydroxy-3-Methoxycinnamaldehyde | 0.00% |
| L-Beta-Homoglutamine-Hcl | 0.00% |
| Citraconic Acid | 0.00% |
| L-Proline | 0.00% |
| 2-Mercaptoethanesulfonic Acid | 0.00% |
| L-Iditol | 0.00% |
| L-Cystine | 0.00% |
| L-Arginine | 0.00% |
| 3'-Methoxy-4',5,7-Trihydroxyflavonol | 0.00% |
| Citrulline | 0.00% |
| Harmaline | 0.00% |
| Citramalate | 0.00% |
| 4-Pyridoxate | 0.00% |
| 2'-Deoxycytidine | 0.00% |
| All-Trans-Retinoic Acid | 0.00% |
| Agmatine | 0.00% |
| 4-Hydroxyphenylpyruvic Acid | 0.00% |
| Guanine | 0.00% |
| Thymidine | 0.00% |
| Glycolaldehyde Dimer,Mixture Of Stereoisomers | 0.00% |
| Triethanolamine | 0.00% |
| (-)-Riboflavin | 0.00% |
| Trans-Cinnamate | 0.01% |
| Cytidine-3',5'-Cyclicmonophosphate | 0.01% |
| Norepinephirine | 0.01% |
| (+-)-Alpha-Tocopherol Acetateacid Ester | 0.01% |
| 4-Nonanolide | 0.01% |
| Dl-Cystathionine | 0.01% |
| L-Gln | 0.01% |
| Dl-Dihydrozeatin | 0.01% |
| Isosakuranetin | 0.01% |
| Paracetamol | 0.01% |
| 3-(4-Hydroxy-3,5-Dimethoxyphenyl)-2-Propenoic Acid | 0.01% |
| His | 0.01% |
| Hinokitiol | 0.01% |
| Taurine | 0.01% |
| Trans-Ortho-Coumaric Acid | 0.01% |
| Thymidine-5'-Monophosphate | 0.01% |
| 1-Methylhistamine | 0.01% |
| Etidronate | 0.01% |
| L-Methionine | 0.01% |
| 1,16-Hexadecanediol | 0.01% |
| Glycyl-L-Proline | 0.01% |
| 5-Butylpyridine-2-Carboxylic Acid | 0.01% |
| Pyridoxamine | 0.01% |
| Alpha-D-Glucose-1,6-Diphosphate | 0.01% |
| Cytidine-5'-Monophosphate | 0.02% |
| Capsaicin | 0.02% |
| S-Methyl-L-Cysteine | 0.02% |
| Gamma-Glu-Cys | 0.02% |
| 3-(4-Hydroxyphenyl)Propionic Acid | 0.02% |
| Cholecalciferol | 0.02% |
| Trans-Zeatin Riboside | 0.02% |
| 3-Methylxanthine | 0.02% |
| N-Carbamoyl-L-Aspartic Acid | 0.02% |
| Kaempferol-3-O-Alpha-L-Rhamnoside | 0.02% |
| Dudp | 0.02% |
| D-(+)-Maltose | 0.02% |
| L-(+)-Lysine | 0.02% |
| 3-(Methylsulfinyl)Propylglucosinolate | 0.03% |
| Mecamylamine | 0.03% |
| Alpha-L-(-)-Fucose 1-Phosphate Bis(Cyclohexylammonium) Salt | 0.03% |
| Phlorizin | 0.03% |
| 2,5-Dihydroxybenzoic Acid | 0.03% |
| Hypoxanthine | 0.03% |
| 2,3-Pyridinedicarboxylic Acid | 0.03% |
| 1,2-Dipalmitoyl-Sn-Glycero-3-Phospho-Rac-(1-Glycerol) Sodium Salt | 0.04% |
| S-Adenosyl-L-Homocysteine | 0.04% |
| Caffeine | 0.04% |
| Baclofen | 0.04% |
| Beta-Nicotinamide Mononucleotide | 0.04% |
| 3-Hydroxy-3-Methylglutaric Acid | 0.04% |
| Cytidine | 0.04% |
| L-Beta-Homomethionine | 0.04% |
| Acacetin-7-O-Rutinoside | 0.04% |
| Phytol | 0.04% |
| Eleutheroside B | 0.05% |
| Guanosine 5'-Diphosphate-D-Mannose | 0.05% |
| Kaempferol-3-O-Alpha-L-Arabinoside | 0.05% |
| Isopentenyladenine | 0.05% |
| L-Saccharopine | 0.05% |
| Glycerophosphate(2) | 0.05% |
| Geranyl Acetone | 0.06% |
| Baicalein-7-O-Glucuronide | 0.06% |
| S-Carboxymethylcysteine | 0.06% |
| Thymidine-3',5'-Cyclic Monophosphate  Sodium Salt | 0.06% |
| 7-Hydroxy-4-Methylcoumarin | 0.07% |
| Cysteine S-Sulfate | 0.07% |
| (+/-)-Cis,Trans-Abscisic Acid | 0.08% |
| 3-Phospho-D-Glycerate | 0.08% |
| (S)-(+)-2-(Anilinomethyl)Pyrrolidine | 0.08% |
| Benzamidine | 0.08% |
| Nicotine | 0.09% |
| Quercetin | 0.10% |
| Cis-Aconitate | 0.10% |
| Nadh | 0.10% |
| Alpha-Tocotrienol | 0.11% |
| N-Octanoylsphingosine,D-Erythro | 0.12% |
| 2-(4-Isobutylphenyl)Propionic Acid | 0.12% |
| 1h-Indole-3-Carboxylic Acid | 0.13% |
| Malvidin-3, 5-Di-O-Glucoside Chloride | 0.13% |
| 5-Aminoimidazole-4-Carboxamide-1-Ribofuranosyl 5'-Monophosphate | 0.13% |
| N-Acetylputrescine | 0.14% |
| Sebacate | 0.14% |
| E-3,4,5'-Trihydroxy-3'-Glucopyranosylstilbene | 0.15% |
| D,L-Sulforaphane | 0.15% |
| N-Palmitoyl-D-Erythro-Sphingosine | 0.15% |
| Cyanidin-3, 5-Di-O-Glucoside | 0.15% |
| D-Cysteine | 0.17% |
| Uridine-5'-Diphosphogalactose Disodium Salt | 0.19% |
| Cyanidin-3-O-(6''-O-(E-P-Coum)-2''-O-(Beta-Xylopyranosyl)-Beta-Glucopyranoside)-5-O-Beta-Glucopyranoside | 0.20% |
| 3,4-Dimethoxycinnamic Acid | 0.20% |
| 3-Chloro-L-Tyrosine | 0.21% |
| Orotic Acid | 0.21% |
| Sissotrin | 0.22% |
| N-Acetyl-L-Cysteine | 0.22% |
| Adenosine 5'-Diphospho-Glucose | 0.22% |
| Pyridoxal 5-Phosphate | 0.23% |
| 3'-Dephosphocoenzyme A | 0.23% |
| Oxypurinol | 0.23% |
| Canthaxanthin | 0.24% |
| Cytidine 5'-Diphosphocholine | 0.25% |
| Quercetin-3-O-Arabinoglucoside | 0.27% |
| Daphnetin | 0.29% |
| Guanosine-5'-Diphosphate Sodium Salt | 0.31% |
| Quercetin-3,4'-O-Di-Beta-Glucopyranoside | 0.32% |
| Tetracosanoic Acid | 0.35% |
| 2'-Deoxyuridine | 0.35% |
| Genistein | 0.37% |
| Glycyrrhizate | 0.38% |
| Adenosine | 0.39% |
| Chlorogenic Acid | 0.39% |
| 1-Myristoyl-2-Hydroxy-Sn-Glycero-3-Phosphoethanolamine | 0.41% |
| Rosmarinic Acid | 0.41% |
| Kaempferol-3-O-(6""-P-Coumaroyl)-Glucoside | 0.41% |
| Benzoic Acid | 0.42% |
| Nicotinamide | 0.45% |
| Cyanidin-3-O-Galactoside | 0.51% |
| 4-Methylsulfinylbutyl Glucosinolate | 0.57% |
| Phenylephrine | 0.58% |
| Uridine 5'-Diphosphoglucuronic Acid | 0.59% |
| Delta-Tocotrienol | 0.59% |
| Gamma-Tocotrienol | 0.62% |
| 3,4-Dihydroxymandelate | 0.66% |
| Spermine | 0.67% |
| Delta-Tocopherol | 0.67% |
| (-)-Alpha-Copaene | 0.68% |
| Peonidin-3,5-O-Di-Beta-Glucopyranoside | 0.73% |
| 1-Myristoyl-2-Hydroxy-Sn-Glycero-3-Phosphate | 0.74% |
| Glucose 6-Phosphate | 0.74% |
| 4-Nitrophenyl Phosphate | 0.75% |
| 2'-Deoxyguanosine 5'-Monophosphate | 0.78% |
| E-4,5'-Dihydroxy-3-Methoxy-3'-Glucopyranosylstilbene | 0.81% |
| Inosine-5'-Diphosphate | 0.84% |
| Sorbitol 6-Phosphate | 0.84% |
| Acacetin | 0.84% |
| N-Acetylglutamate | 0.85% |
| Thiabendazole | 0.87% |
| Rhoifolin | 0.89% |
| Leu-Leu-Tyr | 0.94% |
| Daidzein-8-C-Glucoside | 0.95% |
| 3,5-Dibromo-L-Tyrosine | 0.98% |
| L-Beta-Homoproline | 0.99% |
| Sodium Deoxycholate | 1.00% |
| 1,2-Dilauroyl-Sn-Glycero-3-Phosphate | 1.09% |
| N1-Acetylspermine | 1.11% |
| Isorhamnetin-3-O-Rutinoside | 1.15% |
| Inosine-5'-Triphosphate Trisodium Salt | 1.18% |
| Hyperoside | 1.24% |
| Dl-5-Hydroxylysine | 1.27% |
| Ononin | 1.30% |
| Thymol | 1.32% |
| Quercetin-3-Arabinoside | 1.36% |
| Cyanidin-3-O-(2''-O-Beta-Xylopyranosyl-Beta-Glucopyranoside) | 1.39% |
| Sinapyl Aldehyde | 1.47% |
| S-Lactoylglutathione | 1.48% |
| 2'-Deoxyadenosine-5'-Diphosphate Sodium Salt | 1.59% |
| Hesperetin-7-O-Neohesperidoside | 1.65% |
| Petunidin-3-O-(6''-O-(4'''-O-E-Coum)-Alpha-Rhamnopyranosyl-Beta-Glucopyranosyl)-5-O-Beta-Glucopyranoside Trifluoroacetate Salt | 1.65% |
| Crotonoyl Coenzyme A Lithium Salt | 1.66% |
| Guanosine-5'-Triphosphate Sodium Salt | 1.72% |
| Leupeptin Hemisulfate Salt | 1.78% |
| N-6-(Delta-2-Isopentenyl)Adenosinehemihydrate | 1.88% |
| Pterine | 1.91% |
| 1-O-B-D-Glucopyranosyl Sinapate | 2.02% |
| Eriodictyol-7-O-Glucoside | 2.05% |
| Sarsasapogenin | 2.11% |
| Guanosine | 2.13% |
| Trans-Zeatin | 2.25% |
| N-Benzoyl(D5) Glycine | 2.30% |
| Uridine | 2.34% |
| Linoleic Acid | 2.36% |
| Indole | 2.42% |
| Anserine | 2.44% |
| Piperacillin Sodium Salt | 2.53% |
| Kaempferol-3-O-Robinoside-7-O-Rhamnoside | 2.69% |
| 3-(2-Aminoethyl)Indole | 2.79% |
| Kynurenic Acid | 2.80% |
| Diosmin | 3.05% |
| Adenosine-3',5'-Diphosphate Sodium Salt | 3.18% |
| 7-Acetoxy-4-Methylcoumarin | 3.22% |
| Pelargonidin-3-O-Glucoside | 3.33% |
| Trans-Zeatin-9-Glucoside | 3.47% |
| Suberic Acid | 3.47% |
| A-Lipoamide | 3.62% |
